# GSDME-Dependent Incomplete Pyroptosis Permits Selective IL-1α Release under Caspase-1 Inhibition

**DOI:** 10.1016/j.isci.2020.101070

**Published:** 2020-04-18

**Authors:** Emi Aizawa, Tadayoshi Karasawa, Sachiko Watanabe, Takanori Komada, Hiroaki Kimura, Ryo Kamata, Homare Ito, Erika Hishida, Naoya Yamada, Tadashi Kasahara, Yoshiyuki Mori, Masafumi Takahashi

**Affiliations:** 1 Division of Inflammation Research, Center for Molecular Medicine, Jichi Medical University, 3311-1 Yakushiji, Shimotsuke, Tochigi 329-0498, Japan; 2 Department of Dentistry, Oral and Maxillofacial Surgery, Jichi Medical University, Tochigi, Japan

**Keywords:** Immunology, Cell Biology, Functional Aspects of Cell Biology

## Abstract

Pyroptosis is a form of regulated cell death that is characterized by gasdermin processing and increased membrane permeability. Caspase-1 and caspase-11 have been considered to be essential for gasdermin D processing associated with inflammasome activation. In the present study, we found that NLRP3 inflammasome activation induces delayed necrotic cell death via ASC in caspase-1/11-deficient macrophages. Furthermore, ASC-mediated caspase-8 activation and subsequent gasdermin E processing are necessary for caspase-1-independent necrotic cell death. We define this necrotic cell death as incomplete pyroptosis because IL-1β release, a key feature of pyroptosis, is absent, whereas IL-1α release is induced. Notably, unprocessed pro-IL-1β forms a molecular complex to be retained inside pyroptotic cells. Moreover, incomplete pyroptosis accompanied by IL-1α release is observed under the pharmacological inhibition of caspase-1 with VX765. These findings suggest that caspase-1 inhibition during NLRP3 inflammasome activation modulates forms of cell death and permits the release of IL-1α from dying cells.

## Highlights

• NLRP3 inflammasome induces necrotic cell death in the absence of caspase-1/11
• ASC initiates GSDME-dependent pyroptosis via caspase-8
• IL-1α, but not IL-1β, is released during Casp1/11-independent pyroptosis
• Pharmacological inhibition of caspase-1 permits IL-1α release during pyroptosis

## Graphical Abstract

**Figure:**
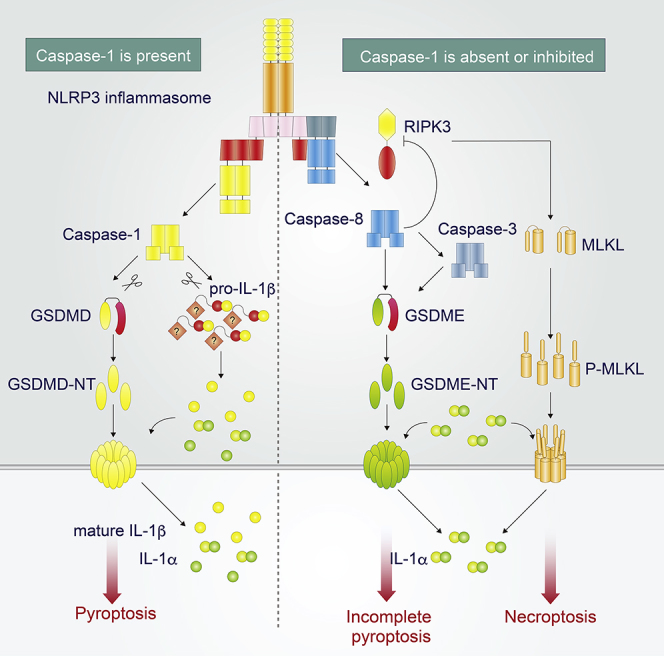

## Introduction

Necrotic cell death associated with various diseases, including myocardial infarction, acute kidney injury, neurodegeneration, and diabetes, triggers inflammatory responses to promote the progression of disease and tissue repair (Tonnus and Linkermann, 2017, Tonnus et al., 2019). Besides accidental necrotic cell death, intrinsic cell mechanisms induce regulated cell death (RCD) to regulate post-injury inflammation (Martin, 2016, Vanden Berghe et al., 2014). Apoptosis, the most extensively studied RCD, is regarded as an anti-inflammatory cell death because it has minimum effects on neighboring cells (Martin et al., 2012). In contrast, necrosis and necrotic RCD can cause inflammation by releasing various damage/danger-associated molecular patterns (DAMPs), such as adenosine triphosphate (ATP), dsDNA, ssRNA, and high-mobility group box 1 (HMGB1), from dying cells (Martin et al., 2012). Thus, regulation of RCD could be a potential target for modulating post-injury inflammation. Meanwhile, the released DAMPs in turn activate pattern recognition receptors (PRRs), which are expressed in innate immune cells and induce subsequent inflammatory responses (Bertheloot and Latz, 2017, Martin et al., 2012). Among PRRs, a group of receptors, including nucleotide-binding oligomerization domain, leucine-rich repeat and pyrin domain containing 3 (NLRP3), NLR and caspase recruitment domain containing 4 (NLRC4), and absent in melanoma 2 (AIM2), form an inflammasome assembly to induce subsequent inflammatory responses through the activation of caspase-1 (Karasawa and Takahashi, 2017, Lamkanfi and Dixit, 2014).

NLRP3 is mainly expressed in innate immune cells such as macrophages and forms “NLRP3 inflammasome” with apoptosis-associated speck-like protein containing a caspase recruitment domain (ASC), which functions as an adaptor protein, and a cysteine proteinase caspase-1 (Karasawa and Takahashi, 2017, Lamkanfi and Dixit, 2014). In response to pathogen-associated molecular patterns (PAMPs) or DAMPs, the components of NLRP3 inflammasome assemble to activate caspase-1, and the active caspase-1 then processes a potent inflammatory cytokine interleukin (IL)-1β into its mature form (Lamkanfi and Dixit, 2014). Similarly, IL-1α, another cytokine belonging to the IL-1 family, is released during NLRP3 inflammasome activation, although it is not directly processed by caspase-1 (Gross et al., 2012). Furthermore, the active caspase-1 induces an inflammatory RCD called pyroptosis (Karasawa and Takahashi, 2017, Lamkanfi and Dixit, 2014).

Among RCDs, pyroptosis is a highly inflammatory cell death that is initiated by inflammatory caspases. Through caspase activation, pyroptotic cells show both apoptotic and necrotic characteristics (Miao et al., 2011). The former includes DNA fragmentation and chromatin condensation, and blebbing. Furthermore, pyroptosis is accompanied by increased membrane permeability, cell swelling, and release of cytosolic content, which is characterized by lactate dehydrogenase (LDH) release. Inflammatory caspases including caspase-1, caspase-4, and caspase-5 have been identified as initiators of pyroptosis in humans. In addition to murine caspase-1, caspase-11, a homolog of caspase-4 and -5 in mice, can initiate pyroptosis. Recent studies have revealed that the executor of pyroptosis mediated by inflammatory caspases is GSDMD (Kayagaki et al., 2015, Shi et al., 2015). GSDMD is a member of the gasdermin family, including GSDMA, B, C, D, E and DFNB59, which share a similar structure (Ding et al., 2016). An amino-terminal domain (NT) of GSDMD possesses pore-forming activity, whereas the carboxy-terminal domain exerts an autoinhibitory effect against NT. After caspase-mediated cleavage of linker domain, NT of GSDMD oligomerizes in plasma membrane where it is enriched with phosphatidylinositol phosphates and forms pores of 13–22 nm (Ding et al., 2016, Liu et al., 2016). The GSDMD-formed pore in turn induces the release of cytosolic content including LDH and IL-1α/β. Since pyroptosis is a highly inflammatory form of cell death (Miao et al., 2011), the modulation of pyroptosis during NLRP3 inflammasome activation is expected to be effective for preventing inflammatory disorders.

The activation of the NLRP3 inflammasome promotes not only caspase-1 activation but also caspase-8 activation as an alternative effector (Antonopoulos et al., 2015) because assembled ASC can be a scaffold for caspase-8 via pyrin domain (Vajjhala et al., 2015). The activated caspase-8 by NLRP3 inflammasome under caspase-1 inhibition is involved in two distinct functions: IL-1β processing and induction of apoptosis via caspase-3 (Antonopoulos et al., 2015). Therefore, inflammasome activation in the absence of caspase-1 induces anti-inflammatory apoptotic cell death (Sagulenko et al., 2013), indicating that caspase-1 inhibition could be a target to convert the form of cell death from pyroptosis to apoptosis. However, other reports suggest that inflammasome activation induces necrotic cell death independent of caspase-1 (Motani et al., 2011, Satoh et al., 2013, Schneider et al., 2017). Thus, the precise mechanisms and the form of cell death induced by NLRP3 inflammasome activation under caspase-1 inhibition remain unclear. In the present study, we characterized necrotic cell death induced by NLRP3 inflammasome activation independent of caspase-1 and -11 (caspase-1/11) and determined that GSDME is involved in this process. We further found that this necrotic cell death is incomplete pyroptosis, which occurs without IL-1β release.

## Results

### Nigericin Induces Caspase-1/11-Independent Necrotic Cell Death via ASC

**Figure 1:**
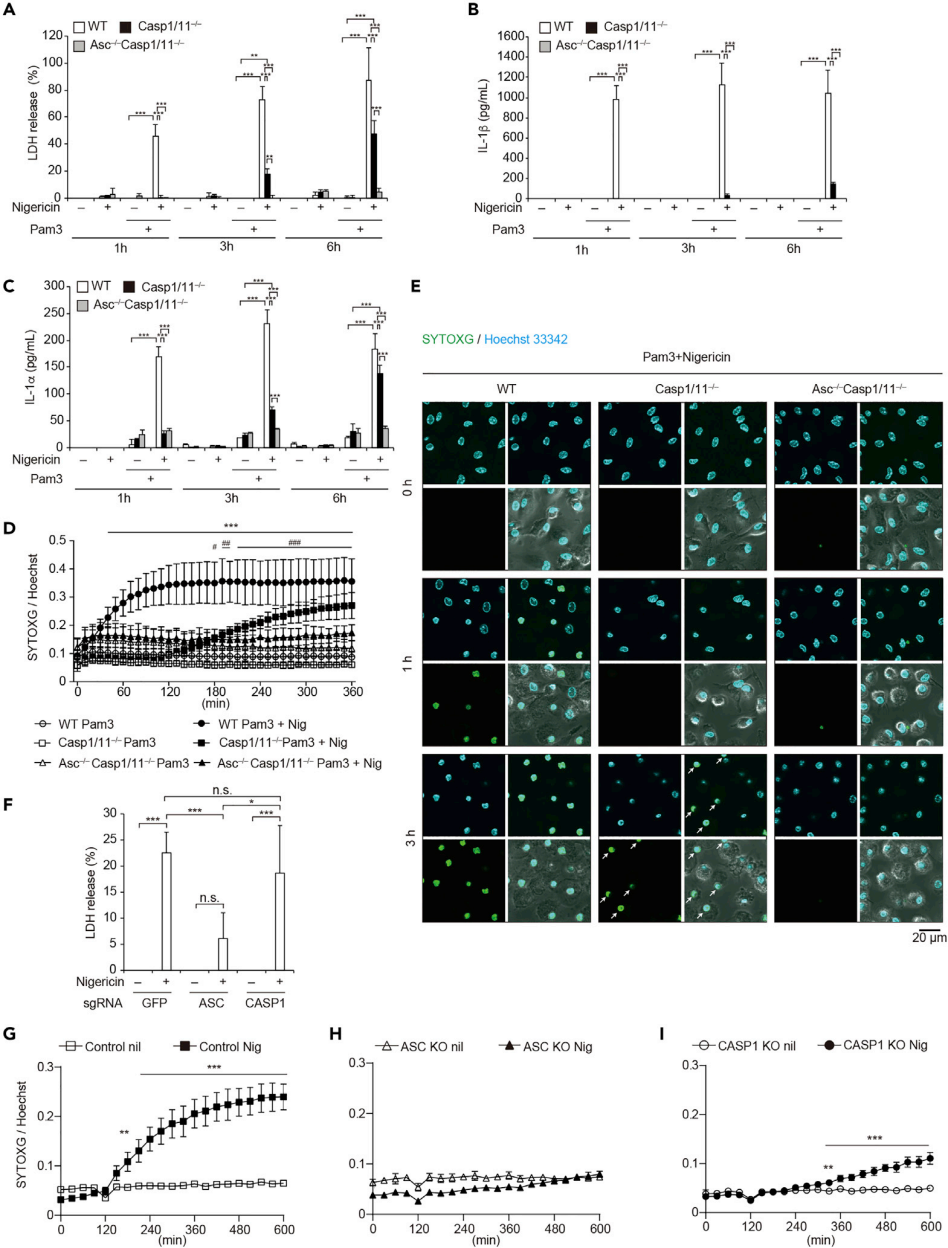
Nigericin Induces Caspase-1/11-Independent Necrotic Cell Death via ASC (A–C) Primary peritoneal macrophages isolated from WT, *Casp1/11*^−/−^, and *Asc*^–/–^*Casp1/11*^−/−^ mice were rested or primed with Pam3CSK4 (100 ng/mL) for 18 h and then treated with nigericin (5 μM) for 1, 3, or 6 h. (A) The levels of LDH in the supernatants were assessed. (B) The levels of IL-1β and (C) IL-1α in the supernatants were assessed by ELISA. (D and E) Primed WT, *Casp1/11*^−/−^, and *Asc*^–/–^*Casp1/11*^−/−^ macrophages were labeled with Hoechst33342 and then treated with nigericin in the presence of SYTOXG. (D) Relative fluorescence units of SYTOXG were measured at 10-min intervals. (E) Images of Hoechst staining (upper left), SYTOXG staining (bottom left), merged images of Hoechst and SYTOXG (upper right), and merged images of fluorescent and bright fields (bottom right) were visualized by confocal microscopy. White arrows indicate dead *Casp1/11*^–/–^ macrophages. (F–I) Control, *ASC* KO, and *CASP1* KO THP-1 cells were differentiated with PMA for 48 h and then treated with nigericin (5 μM). (F) The levels of LDH in the supernatants 8 h after nigericin stimulation were assessed. (G) Relative fluorescence units of SYTOXG in (G) Control, (H), *ASC* KO, (I) and *CASP1*KO THP1 cells were measured at 30-min intervals. Data represent mean ± SD of three (A and D) or four (F) independent experiments. (B, C, and G–I) Data are shown as mean ± SD of triplicate of one experiment. (B, C, E, and G–I) Data are representative of two independent experiments. (A–C, F, G, I) ∗p < 0.05, ∗∗p < 0.01, ∗∗∗p < 0.001 as determined by two-way ANOVA with a post hoc test. (D) ∗∗∗p < 0.001 compared with WT Pam3 and WT Pam3+Nig, #p < 0.05, ##p < 0.01, ###p < 0.001 compared with *Casp1/11*^–/–^ Pam3 and *Casp1/11*^–/–^ Pam3+Nig as determined by two-way ANOVA with a post hoc test. n.s., not significant.

To investigate whether NLRP3 inflammasome activation induces necrotic cell death independent of caspase-1/11, wild-type (WT), NLRP3 knockout (*Nlrp3*^−/−^), or caspase-1 and caspase-11 double-knockout (*Casp1*/*11*^−/−^) peritoneal macrophages were primed with Pam3CSK4 for 18 h and then stimulated with nigericin, a potent NLRP3 inflammasome activator. LDH release was induced immediately after nigericin stimulation in WT macrophages, whereas the response was inhibited in both *Nlrp3*^*−/−*^ and *Casp1/11*^*−/−*^ macrophages (Figures S1A and S1B). At later time point, however, LDH release was detected from *Casp1/11*^−/−^ macrophages, indicating that NLRP3 inflammasome induces caspase-1/11-independent cell death. Since ASC is an essential scaffold for signal transduction mediated by NLRP3 inflammasome (Lamkanfi and Dixit, 2014), *Casp1/11*^−/−^ mice were crossed with ASC knockout (*Asc*^–/–^) mice to investigate the role of ASC in caspase-1/11-independent necrotic cell death. Indeed, nigericin-induced LDH release at later time point was canceled in *Asc*^*–*/–^ *Casp1/11*^−/−^ macrophages (Figure 1A). As expected, IL-1β release was completely inhibited from both *Casp1/11*^−/−^ macrophages and *Asc*^–/–^ *Casp1/11*^−/−^ macrophages even at a later time point (Figure 1B). Notably, a substantial amount of IL-1α release was detected from *Casp1/11*^−/−^ macrophages, whereas IL-1α release was also canceled in *Asc*^*–*/–^ *Casp1/11*^−/−^ macrophages (Figure 1C). To investigate the precise time course of nigericin-induced necrotic cell death in *Casp1/11*^−/−^ macrophages, we used SYTOX green (SYTOXG) staining assay with real-time monitoring of dead cells. In WT macrophages, SYTOXG fluorescence started to increase 40 min after nigericin stimulation (Figure 1D). On the other hand, cell death in *Casp1/11*^−/−^ macrophages began 180 min after stimulation. This delayed cell death was also visualized by confocal microscopy (Figure 1E, indicated by white arrows). Although cell swelling, a key morphological change during pyroptosis, was observed in both WT and *Casp1/11*^−/−^ macrophages, nuclei of *Casp1/11*^−/−^ macrophages were more condensed than those of WT macrophages (Figures S1C–S1E). Although we tested the effect of priming duration on caspase-1-independent necrotic cell death, the capability of the necrotic cell death was reduced in *Casp1/11*^−/−^ macrophages primed with Pam3CSK4 for 4 h (Figures S2A–S2E). Next, we assessed whether similar responses could be observed in other types of macrophages and confirmed that caspase-1/11-independent necrotic cell death was observed in bone marrow-derived macrophages (Figure S2F). Caspase-1-independent necrotic cell death was also assessed in THP-1 cells, which are a human monocytic cell line that can differentiate into macrophages. Similar to murine macrophages, nigericin-induced LDH release was observed in *CASP1* KO THP1 cells, but not in *ASC* KO THP-1 cells (Figures 1F and S2G). Similar trends were confirmed by an SYTOXG assay (Figures 1G–1I, S1F, and S1G). Unlike nigericin, lysosome-damaging stimuli such as cholesterol crystals, palmitic acid crystals, and nanosilica particles induced necrotic cell death in both *CASP1* KO and *ASC* KO THP-1 cells (Figure S2H).

### NLRP3 Inflammasome Activation Induces Necrotic Cell Death in the Absence of Caspase-1

**Figure 2:**
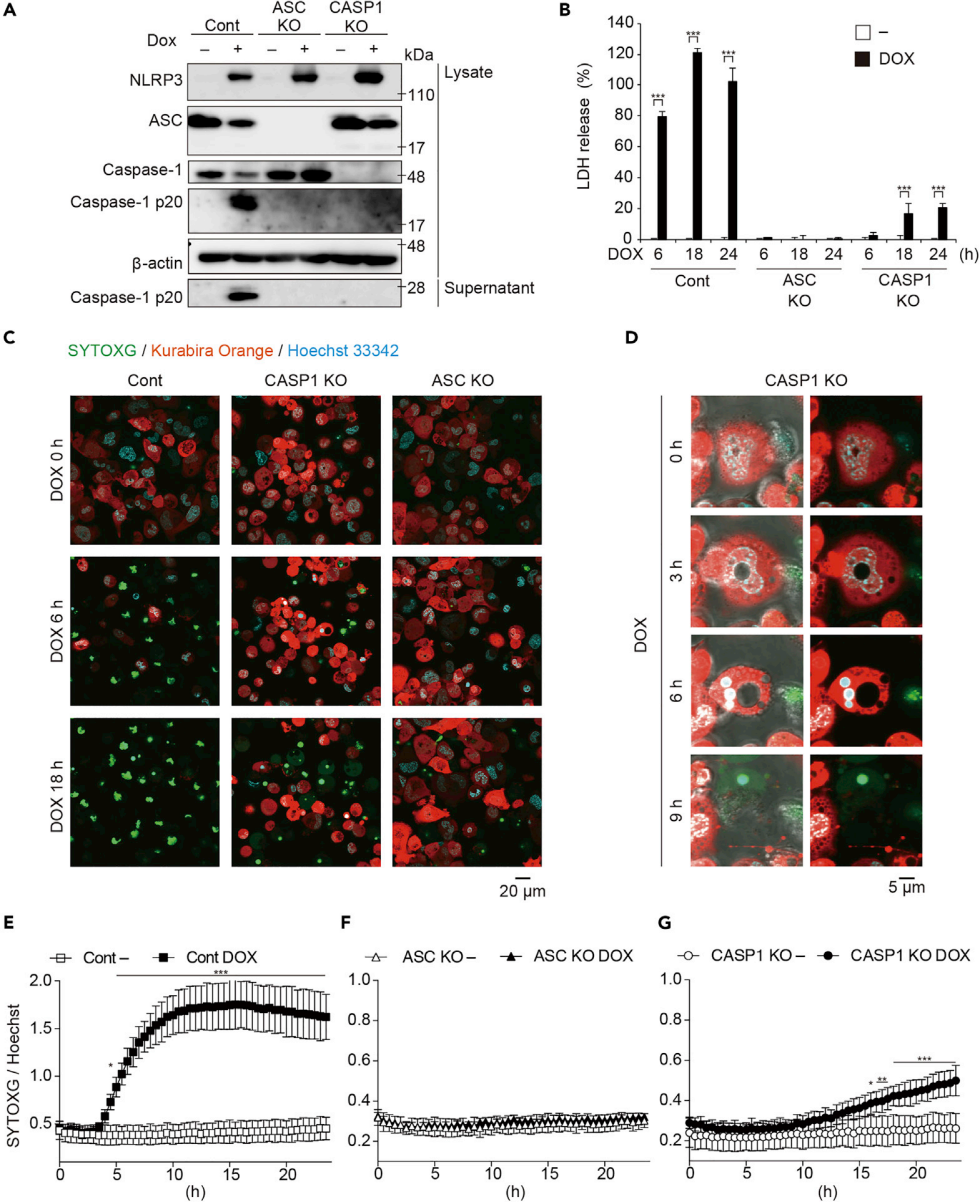
NLRP3 Inflammasome Activation Induces Necrotic Cell Death in the Absence of Caspase-1 (A–F) Control, *ASC* KO, and *CASP1*KO THP1 *NLRP3 D303N* cells were differentiated with PMA for 48 h and then treated with DOX (1 μg/mL). (A) After 6 h, lysates and supernatants were analyzed by western blot. (B) The levels of LDH in the supernatants at the indicated time points were assessed. (C and D) Cells were treated with DOX in the presence of SYTOXG. (C) Merged images of hKO1, SYTOXG, and Hoechst33342 were visualized by confocal microscopy. (D) High-magnification images of DOX-treated *CASP1*KO THP1 *NLRP3 D303N* cells. Images were visualized as merged images of fluorescence (right panels) and merged images of fluorescence and bright fields (left panels). (E–G) Relative fluorescence units of SYTOXG in (E) Control, (F) *ASC* KO, and (G) *CASP1*KO THP1 *NLRP3 D303N* cells were measured at 30-min intervals. Data are shown as mean ± SD of triplicate (B) or pentaplicate (E–G) of one experiment. (A–G) Data are representative of two independent experiments. ∗p < 0.05, ∗∗p < 0.01, ∗∗∗p < 0.001 as determined by two-way ANOVA with a post hoc test.

To exclude the possibility that nigericin exerted non-specific cytotoxicity, we developed THP-1 cells expressing a NLRP3D303N mutant under the TET-ON promoter (*NLRP3D303N*-THP-1 cells; Figure S3A). NLRP3D303N is a causative mutation of cryopyrin-associated periodic syndromes and is constitutively active. Indeed, doxycycline (DOX)-induced NLRP3D303N expression led to subsequent release of LDH and IL-1β (Figures S3B–S3D). Increased membrane permeability by NLRP3 inflammasome activation was further confirmed by flow cytometry analysis with annexin V and 7-AAD staining (Figure S3E). Next, we produced *ASC* KO or *CASP1* KO *NLRP3D303N* -THP-1 cells. DOX-mediated inflammasome activation characterized by caspase-1 activation was prevented in both *ASC* KO and *CASP1* KO THP-1 cells (Figure 2A). Similar to nigericin-induced LDH release, inflammasome activation-mediated delayed LDH release was detected in *CASP1* KO cells, whereas LDH release was completely prevented in *ASC* KO cells (Figure 2B). To visualize a loss of cytosolic content during pyroptosis, the developed *NLRP3D303N* -THP-1 cells also expressed fluorescent humanized Kusabira Orange (hKO1) protein (Figure S3A). A loss of cytosolic contents and increased membrane permeability during pyroptosis in control cells were successfully visualized with hKO1 and SYTOXG (Figures 2C and S3F). The delayed necrotic cell death in *CASP1* KO cells was also confirmed by loss of hKO1 and SYTOXG staining 18 h after DOX treatment. In addition, necrotic cell death in *CASP1*KO cells was brought about after apoptosis-like morphological changes such as nuclear condensation and shrinkage (Figures 2D and S3G). Furthermore, the onset of cell death was determined using SYTOXG. In control cells, cell death as indicated by SYTOXG fluorescence was increased 4 h after DOX treatment (Figure 2E). Consistent with the LDH assay, SYTOXG fluorescence was significantly increased 16 h after DOX treatment in *CASP1* KO cells, whereas it was unchanged in *ASC* KO cells (Figures 2F and 2G).

### Other Caspases Are Involved in Caspase-1/11-Independent Necrotic Cell Death Induced by NLRP3 Inflammasome Activation

**Figure 3:**
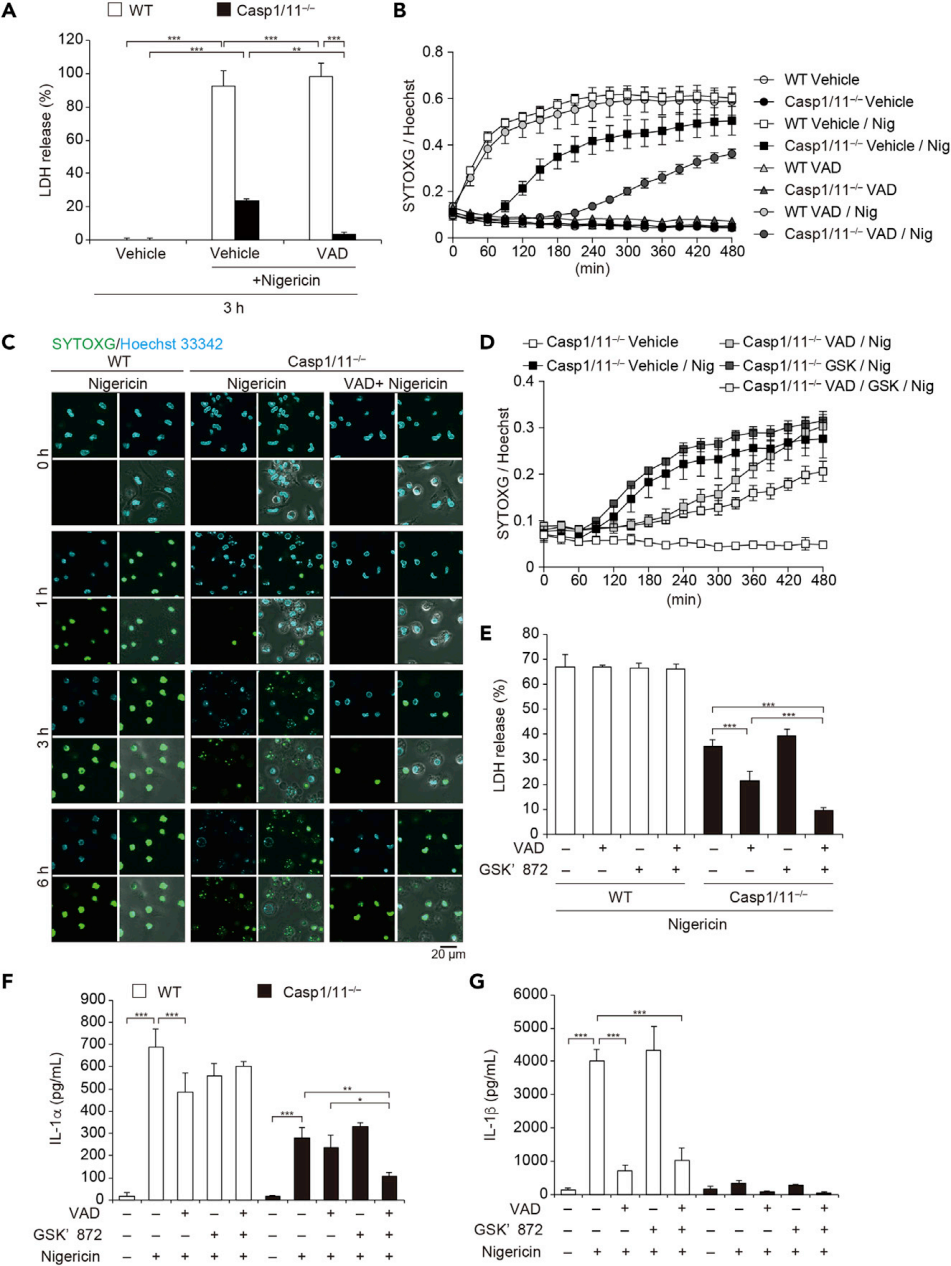
Other Caspases Are Involved in Caspase-1/11-Independent Necrotic Cell Induced by NLRP3 Inflammasome Activation (A–C) Pam3CSK4-primed WT and *Casp1/11*^−/−^ macrophages were pretreated with Z-VAD (20 μM) and then treated with nigericin (5 μM). (A) The levels of LDH in the supernatants were assessed 3 h after nigericin treatment. (B) Relative fluorescence units of SYTOXG were measured at 30-min intervals. (C) Images were visualized by confocal microscopy. (D–G) Pam3CSK4-primed WT and *Casp1/11*^−/−^ macrophages were pretreated with Z-VAD and GSK′872 (3 μM) and then treated with nigericin. (D) Relative fluorescence units of SYTOXG were measured at 30-min intervals. (E) The levels of LDH in the supernatants were assessed. (F) The levels of IL-1α and (G) IL-1β in the supernatants were assessed by ELISA. (A, B, and D–F) Data are shown as mean ± SD of triplicate of one experiment. Data are representative of two (B, C, F, and G) or three (A, D, and E) independent experiments. ∗p < 0.05, ∗∗p < 0.01, ∗∗∗p < 0.001 as determined by two-way ANOVA with a post hoc test.

Caspase-8 functions as an initiator caspase in *Casp1/11*^*−/−*^ cells during NLRP3 inflammasome activation (Antonopoulos et al., 2015). Thus, we postulated that inhibition of other caspases could prevent caspase-1/11-independent necrotic cell death induced by NLRP3 inflammasome activation. Indeed, NLRP3 inflammasome-mediated necrotic cell death in the absence of caspase-1 was prevented by a pan-caspase inhibitor Z-VAD (Figures 3A and S4A–S4C). Dead cell monitoring with SYTOXG revealed that the onset of cell death was delayed by Z-VAD treatment in *Casp1/11*^−/−^ macrophages (Figure 3B). This delayed cell death was also confirmed by confocal microscopy (Figure 3C). However, necrotic cell death was detected even in Z-VAD-treated *Casp1/11*^−/−^ macrophages at a later time point (Figures 3B and S4D). Since caspase-8 inactivates receptor-interacting serine-threonine kinase 3 (RIPK3) to prevent necroptosis, the induction of necroptosis in VAD-treated cells is possible (Vanden Berghe et al., 2014). To assess the involvement of necroptosis in this necrotic cell death, a RIPK3 inhibitor GSK′872 was used together with Z-VAD. Combined treatment with Z-VAD and GSK′872 significantly inhibited LDH release and delayed the onset of cell death compared with Z-VAD treatment in *Casp1/11*^−/−^ macrophages (Figures 3D and 3E). In agreement with LDH release, IL-1α release in *Casp1/11*^−/−^ macrophages was substantially induced, and this induction was inhibited by combined treatment with Z-VAD and GSK′872 (Figure 3F). In contrast, IL-1β release was not detected in *Casp1/11* macrophages (Figure 3G). These results suggest that caspase-1/11-independent necrotic cell death induced by NLRP3 inflammasome activation was mediated by caspase-dependent pyroptosis. Furthermore, RIPK3-dependent necroptosis occurred instead of pyroptosis when caspases were inhibited.

### GSDME Is Processed in Caspase-1/11-Independent Pyroptosis Induced by NLRP3 Inflammasome Activation

**Figure 4:**
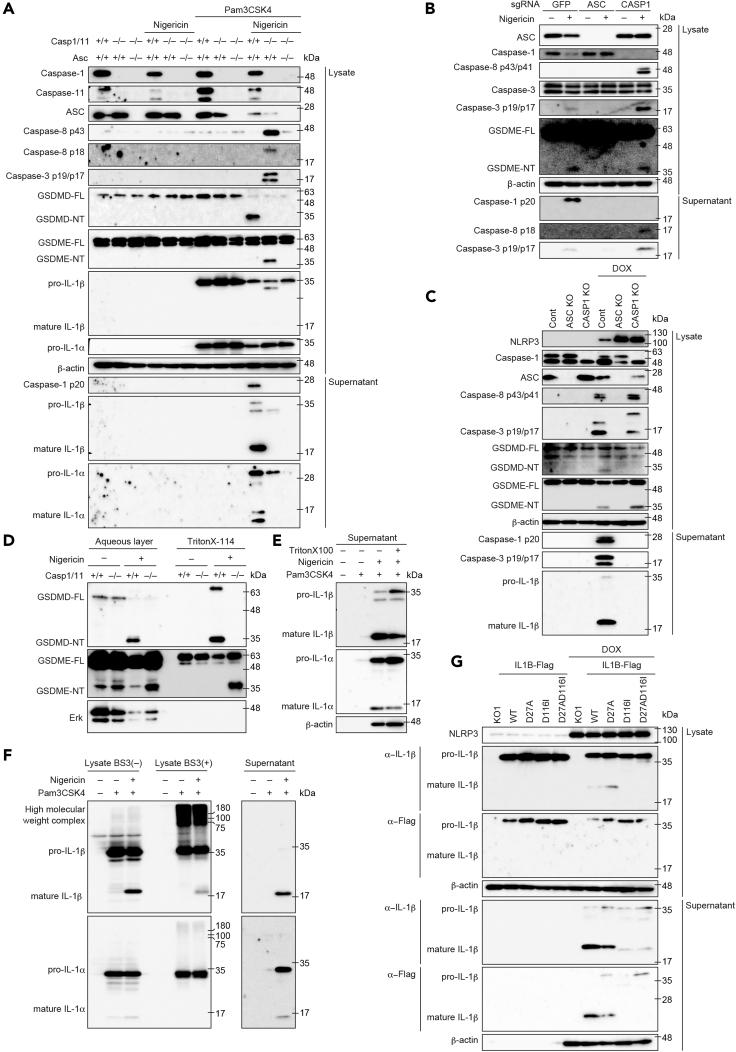
GSDME Is Processed in Caspase-1 Caspase-1/11-Independent Necrotic Cell Induced by NLRP3 Inflammasome Activation (A) Primary peritoneal macrophages isolated from WT, *Casp1/11*^−/−^, and *Asc*^*–*/–^*Casp1/11*^−/−^ mice were rested or primed with Pam3CSK4 (100 ng/mL) for 18 h and then treated with nigericin (5μM) for 3 h. Lysates and supernatants were analyzed by western blot. (B) Control, *ASC*KO, and *CASP1* KO THP1 cells were differentiated with PMA for 48 h and treated with nigericin (5 μM) for 8 h. Lysates and supernatants were analyzed by western blot. (C) Control, *ASC*KO, and *CASP1*KO THP1 *NLRP3 D303N* cells were differentiated with PMA for 48 h and treated with DOX (1 μg/mL) for 18 h. Lysates and supernatants were analyzed by western blot. (D) Primed WT and *Casp1/11*^−/−^ macrophages were treated with nigericin for 3 h. Cells were lysed with Triton X-114 and separated into an aqueous phase and detergent phase. Each fraction was precipitated by acetone and analyzed by western blot. (E and F) Pam3CSK4-primed WT macrophages were stimulated with nigericin. (E) After 3 h, cells were lysed with Triton X and supernatants were analyzed by western blot. (F) After 30 min, cell lysates were cross-linked with BS3 and analyzed by western blot. (G) THP1 *NLRP3 D303N*/*hIL1B* cells were differentiated with PMA for 48 h and treated with DOX for 6 h. Lysates and supernatants were analyzed by western blot. (A–G) Data are representative of two independent experiments.

To identify the molecule that is responsible for nigericin-induced necrotic cell death in *Casp1/11*^−/−^ macrophages, we assessed the processing of caspases and gasdermins. In primed WT macrophages, nigericin induced caspase-1 activation and subsequent GSDMD processing that were completely prevented in *Casp1/11*^−/−^ macrophages (Figure 4A). By contrast, caspase-8 and -3 (caspase-8/3) were activated in nigericin-treated primed *Casp1/11*^−/−^ macrophages. Recently, Wang et al. demonstrated that another gasdermin, GSDME/DFNA5, could be processed by caspase-3 and induce pyroptosis (Wang et al., 2017). Indeed, GSDME was apparently processed only in stimulated *Casp1/11*^−/−^ macrophages, whereas neither GSDMD-NT nor GSDME-NT was observed in *Asc*^*–*/–^*Casp1/11*^−/−^ macrophages (Figure 4A). Similarly, the processing of GSDME accompanied by caspase-8/3 activation was induced in nigericin-stimulated *CASP1* KO THP-1 cells and DOX-treated *CASP1* KO *NLRP3D303N*-THP-1 cells (Figures 4B and 4C). Next, we assessed the time course of GSDME processing because the onset of nigericin-induced necrotic cell death in *Casp1/11*^−/−^ macrophages was slower than that in WT macrophages. In agreement with the delayed onset of cell death, processing of GSDME strongly proceeded 1–3 h after stimulation (Figure S5A). Previous studies have suggested that NT of gasdermins binds phosphatidylinositol and translocates to plasma membrane during pyroptosis (Ding et al., 2016, Liu et al., 2016). Therefore, we assessed whether GSDMD-NT and GSDME-NT could be located in Triton X-114-soluble membrane-containing fraction. Full-length GSDMD and GSDME were distributed in the aqueous phase (Figure 4D). Although processed GSDMD-NT was detected in the Triton X-114-soluble phase in nigericin-treated WT macrophages, GSDMD-NT was not found in the same fraction of nigericin-treated *Casp1/11*^−/−^ macrophages. Instead, GSDME-NT was distributed in Triton X-114-soluble phase in nigericin-treated *Casp1/11*^−/−^ macrophages. These results indicate that distinct gasdermins were processed for the induction of pyroptosis in the presence or absence of caspase-1/11 during NLRP3 inflammasome activation.

### Unprocessed pro-IL-1β Is Retained in Pyroptotic Cells under Caspase-1 Inhibition

Previous reports suggested that IL-1β can be processed by caspase-8 even in the absence of caspase-1 (Antonopoulos et al., 2015, Schneider et al., 2017). However, we found that mature IL-1β was only faintly detected in the lysate of stimulated *Casp1/11*^−/−^ macrophages despite substantial activation of caspase-8 (Figure 4A). Instead, partially processed IL-1β (estimated molecular weight 28 kDa) was detected in lysates and supernatants of nigericin-stimulated *Casp1/11*^−/−^ macrophages, indicating that mature IL-1β (p17) was not a major product of caspase-8-mediated processing. Meanwhile, although only a modest amount of pro-IL-1β was detected in supernatants of nigericin-stimulated WT macrophages, substantial amounts of mature IL-1β and IL-1α were detected in the same fraction. Therefore, we assumed that some unidentified mechanism holds unprocessed pro-IL-1β in pyroptotic cells. To assess the remaining pro-IL-1β in pyroptotic cells, WT macrophages that had undergone pyroptosis 3 h after nigericin stimulation were lysed by Triton X-100. Mature IL-1β in supernatants was not increased by cell lysis, whereas pro-IL-1β in supernatants was dramatically increased by treatment with Triton X-100 (Figure 4E). To assess the possibility that pro-IL-1β forms a molecular complex that is retained in the cytosol, a cross-link analysis with bis (sulfosuccinimidyl) suberate, disodium salt (BS3) was performed. IL-1β was detected as a higher-molecular-weight complex when reacted with BS3, whereas most IL-1α was detected as a monomer (Figure 4F). To determine whether caspase-mediated processing is needed for IL-1β release, we constructed uncleavable human IL-1β mutants by replacing Asp 27 and Asp116, which are reported to be cleavage sites of pro-IL-1β (Figure S5B). Next, lentiviral vectors encoding WT and mutated IL-1β were transduced into *NLRP3D303N*-THP-1 cells. Consistent with the results with peritoneal macrophages, selective release of mature IL-1β was detected during pyroptosis induced by NLRP3D303N (Figures 4G and S5C). As expected, mutated IL-1βD116I and D27A/D116I were not cleaved during NLRP3 inflammasome activation (Figure 4G). Moreover, mutated pro-IL-1βD116I and D27A/D116I were faintly detected in the supernatant after pyroptosis, whereas processed WT IL-1β was clearly detected in the supernatant. The mutated pro-IL-1βD116I and D27A/D116I were retained in the pyroptotic cells because treatment with Triton X-100 increased mutated pro-IL-1β in supernatants (Figure S5D). Taken together, these results suggest that pro-IL-1β forms an intracellular molecular complex and caspase-mediated processing is required for efficient IL-1β release from pyroptotic cells.

### Caspase-8 Initiates GSDME Processing during NLRP3 Inflammasome Activation

**Figure 5:**
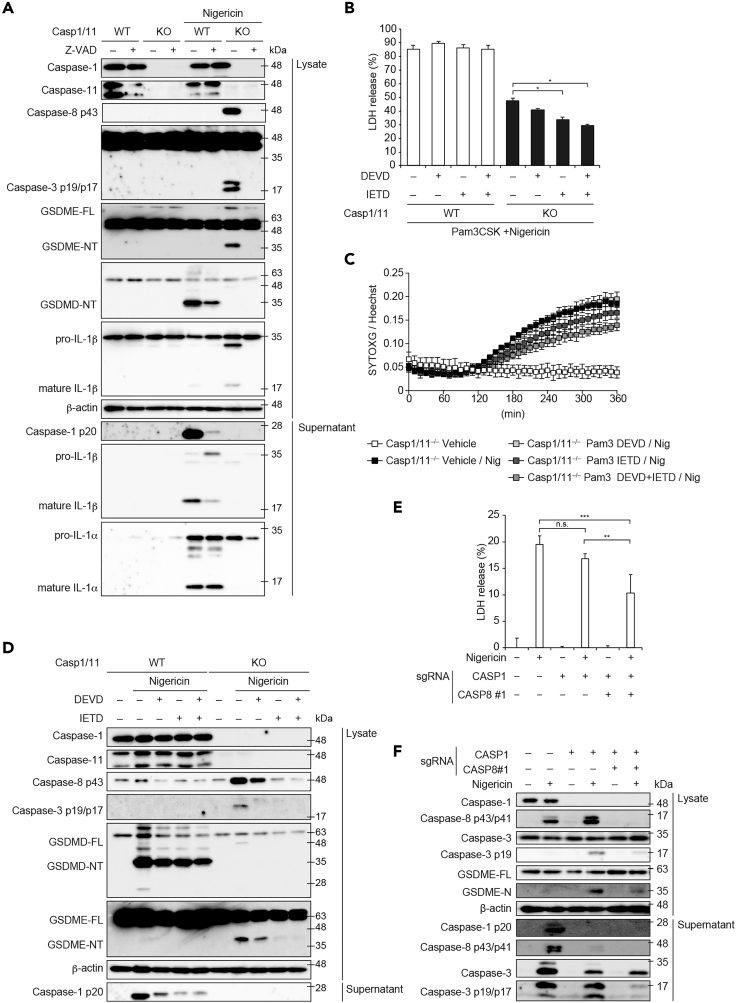
Caspase-8 Initiates Gasdermin E Processing during NLRP3 Inflammasome Activation (A) Pam3CSK4-primed WT and *Casp1/11*^−/−^ macrophages were pretreated with Z-VAD (20 μM) and then treated with nigericin (5 μM) for 3 h. Lysates and supernatants were analyzed by western blot. (B–D) Pam3CSK4-primed WT and *Casp1/11*^−/−^ macrophages were pretreated with DEVD and IETD (20 μM each) and then treated with nigericin. (B) After 6 h, the levels of LDH in the supernatants were assessed. (C) Relative fluorescence units of SYTOXG were measured at 10-min intervals. (D) After 3 h, lysates and supernatants were analyzed by western blot. (E and F) Control, *CASP1* KO, and *CASP1* and *CASP8* double-KO THP1 cells were differentiated with PMA for 48 h and then treated with nigericin (5 μM) for 8 h (E) LDH release in supernatant was assessed. (F) Lysates and supernatants were analyzed by western blot. (B, C, and E) Data are shown as mean ± SD of triplicate of one experiment. Data are representative of two (A and D–F) or three (B and C) independent experiments. ∗p < 0.05, ∗∗p < 0.01, ∗∗∗p < 0.001 as determined by two-way ANOVA with a post hoc test. n.s., not significant.

Our results suggest that *Casp1/11*-independent pyroptosis during NLRP3 inflammasome activation is mediated by caspase-dependent GSDME processing. Therefore, we assessed whether Z-VAD could inhibit GSDME processing in *Casp1/11*^−/−^ macrophages. Z-VAD-treatment inhibited the activation of caspases including caspase-1 in WT and caspase-8/3 in Casp1/11^−/−^ macrophages, respectively (Figure 5A). Reportedly, the processing of GSDMD in WT macrophages was partially inhibited by Z-VAD-treatment (Schneider et al., 2017). In contrast, the processing of GSDME in Casp1/11^−/−^ macrophages was completely abolished by Z-VAD-treatment. Because nigericin induced RIPK3-dependent necrotic cell death in Z-VAD-treated Casp1/11^−/−^ macrophages (Figures 3D and 3E), we analyzed signaling of necroptosis. Nigericin stimulation promoted insoluble complex formation not only of ASC but also of RIPK3 in primed Casp1/11^−/−^ macrophages (Figure S5E). The accumulation and phosphorylation of RIPK3 in insoluble fraction were further enhanced by Z-VAD treatment. Concordantly, the phosphorylation of mixed lineage kinase domain like pseudokinase (MLKL) was enhanced under Z-VAD treatment (Figure S5F). These results suggest that inflammasome activation causes MLKL-mediated necroptosis instead of gasdermin-dependent pyroptosis only when caspase-1 and caspase-8 are inhibited. To further identify the caspases that are responsible for GSDME processing, specific inhibitors of caspases were used. Although both a caspase-3 inhibitor DEVD and a caspase-8 inhibitor IETD inhibited LDH release in nigericin-treated *Casp1/11*^−/−^ macrophages, the inhibitory effect of IETD was greater than that of DEVD (Figure 5B). This finding was also confirmed by an SYTOXG assay (Figure 5C). In accordance with the inhibitory effect on cell death, the processing of GSDME was abolished only in IETD-treated-*Casp1/11*^−/−^ macrophages (Figure 5D). To further assess the impact of caspase-8 on caspase-1-independent necrotic cell death, we established *CASP1* and *CASP8* double-KO THP1 cells (*CASP1*/*CASP8* DKO cells) (Figure S6A). LDH release, caspase-3 activation, and GSDME processing were attenuated in *CASP1*/*CASP8* DKO cells compared with those in *CASP1* KO cells (Figures 5E and 5F). These results suggest that caspase-8 initiates GSDME processing in caspase-1-independent pyroptosis.

### GSDME Serves as an Alternative Gasdermin in NLRP3 Inflammasome Activation

**Figure 6:**
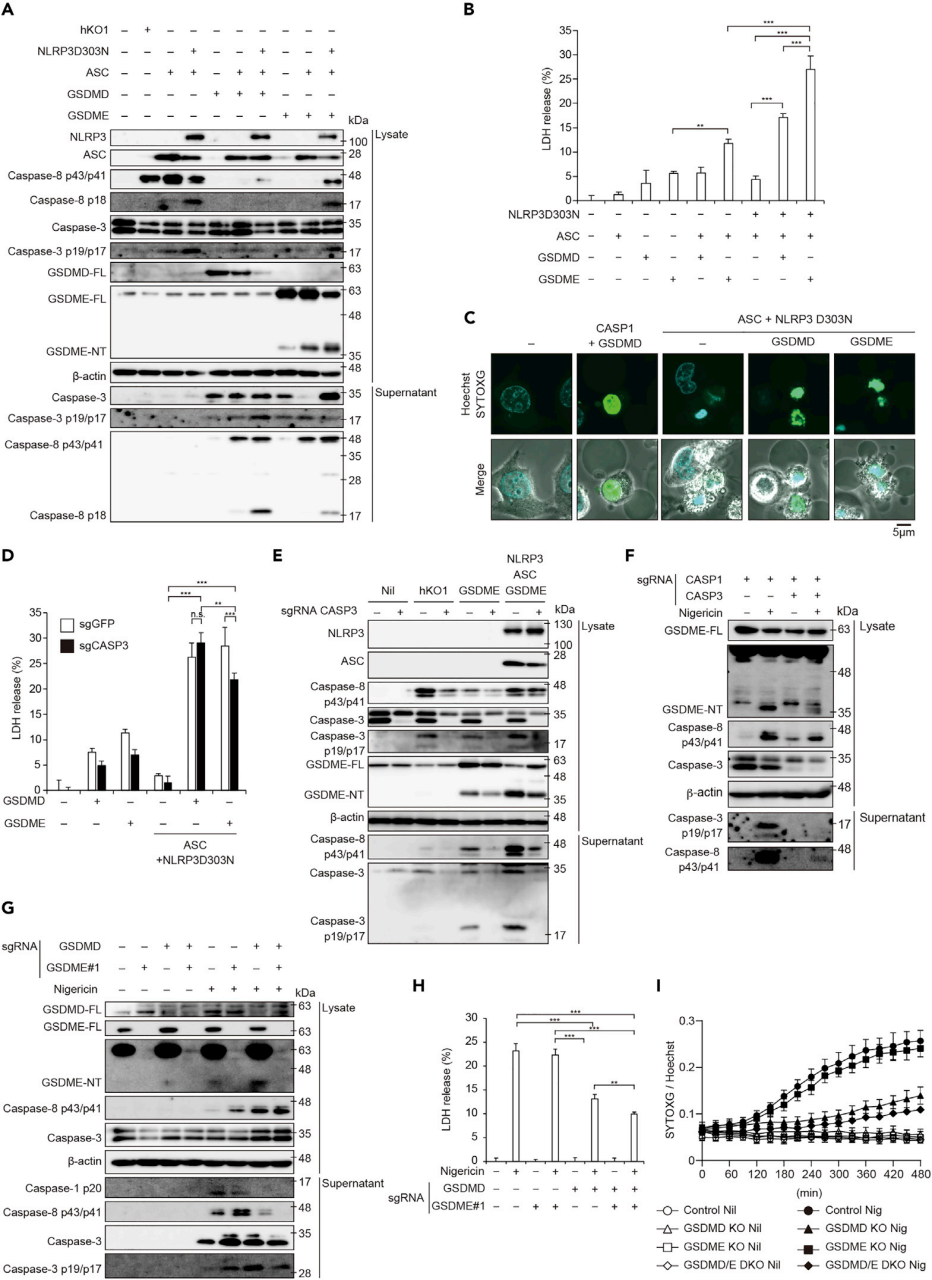
GSDME Serves as an Alternative Gasdermin in NLRP3 Inflammasome Activation (A–C) HeLa cells were transfected with indicated plasmids and cultured for 24 h. (A) Lysates and supernatants were analyzed by western blot. (B) LDH release in supernatant was assessed. (C) Cells were stained with SYTOXG and Hoechst33342 and analyzed by confocal microscopy. (D and E) *CASP3* KO HeLa cells were transiently transfected. (D) LDH release in supernatant was assessed. (E) Lysates and supernatants were analyzed by western blot. (F) *CASP1* KO and *CASP1* and *CASP3* double-KO THP1 cells were differentiated with PMA for 48 h and then treated with nigericin (5 μM) for 8 h. Lysates and supernatants were analyzed by western blot. (G–I) Control, *GSDMD* KO, *GSDME* KO, and *GSDMD* and *GSDME* double-KO THP1 cells were differentiated with PMA for 48 h and treated with nigericin (5 μM) for 8 h. (G) Lysates and supernatants were analyzed by western blot. (H) LDH release in supernatant was assessed. (I) Relative fluorescence units of SYTOXG were measured at 30-min intervals. (B, D, H, and I) Data are shown as mean ± SD of triplicate of one experiment. (A–H) Data are representative of two independent experiments. ∗∗p < 0.01, ∗∗∗p < 0.001 as determined by two-way ANOVA with a post hoc test.

Next, we investigated whether NLRP3 inflammasome induces GSDME processing and subsequent pyroptosis in the absence of caspase-1. To this end, HeLa cells, which lack caspase-1 but express caspase-8 (Figure S6B), were transiently transfected with NLRP3 D303N, ASC, GSDMD, and GSDME. Reconstitution of constitutively active NLRP3 inflammasome induced caspase-8 activation, GSDME processing, and subsequent LDH release (Figures 6A and 6B). Cells reconstituted with active NLRP3 inflammasome exhibited an apoptosis-like morphology with cell shrinkage and chromatin condensation, whereas coexpression of active NLRP3 inflammasome with GSDME induced a pyroptosis-like morphology with cell swelling (Figure 6C). Since caspase-8 reportedly processes GSDMD (Orning et al., 2018, Sarhan et al., 2018), we also compared the processing of FLAG-tagged GSDMD and GSDME induced by ASC-mediated caspase-8 activation. Consistent with our findings in *Casp1/11*^−/−^ macrophages, ASC-mediated caspase-8 activation preferentially promoted the processing of GSDME compared with GSDMD (Figure S6C), suggesting that NLRP3 inflammasome activation induces caspase-1-independent pyroptosis via GSDME processing. On the other hand, previous studies have suggested that caspase-3 is a responsible enzyme for GSDME processing (Rogers et al., 2017, Wang et al., 2017). To examine the role of caspase-3 in GSDME processing, we established *CASP3* KO HeLa cells (Figure S6D). When NLRP3 inflammasome was expressed with GSDME ectopically, LDH release from *CASP3* KO cells was partially attenuated compared with that from control cells (Figure 6D). Although *CASP3* deficiency attenuated GSDME processing, GSDME-NT were still detected even in the absence of caspase-3 (Figure 6E). In order to further analyze caspase-3-independent GSMDE processing by inflammasome activation, we developed *CASP1* and *CASP3* double-KO THP1 cells (*CASP1*/*CASP3* DKO cells) (Figure S6E). Nigericin-mediated inflammasome activation induced modest processing of GSDME accompanied by caspase-8 activation in *CASP1*/*CASP3* DKO cells (Figure 6F). In accordance with GSDME processing, nigericin-induced necrotic cell death in *CASP1*/*CASP3* DKO cells. (Figures S6F and S6G). These results suggest that caspase-3 contributes to, but is not indispensable for, GSDME processing.

To determine the contribution of GSDME as an alternative gasdermin to the induction of pyroptosis, we established *GSDMD* and *GSDME* DKO (*GSDMD*/*E* DKO cells) THP1 cells (Figure S6H). Caspase-8/3 activation and subsequent GSDME processing were increased in nigericin-stimulated *GSDMD* KO cells compared with those in nigericin-stimulated control cells (Figure 6G). Nigericin-induced increased membrane permeability and LDH release in *GSDMD*/*E* DKO cells were attenuated compared with those in *GSDMD* KO cells (Figures 6H and 6I), indicating that GSDME partially contributes to pyroptosis in the absence of GSDMD. These results suggest that not only GSDMD but also GSDME initiates pyroptosis via caspase-8 activation.

### Pharmacological Inhibition of Caspase-1 Activation Dissociates IL-1β Release and Pyroptosis

**Figure 7:**
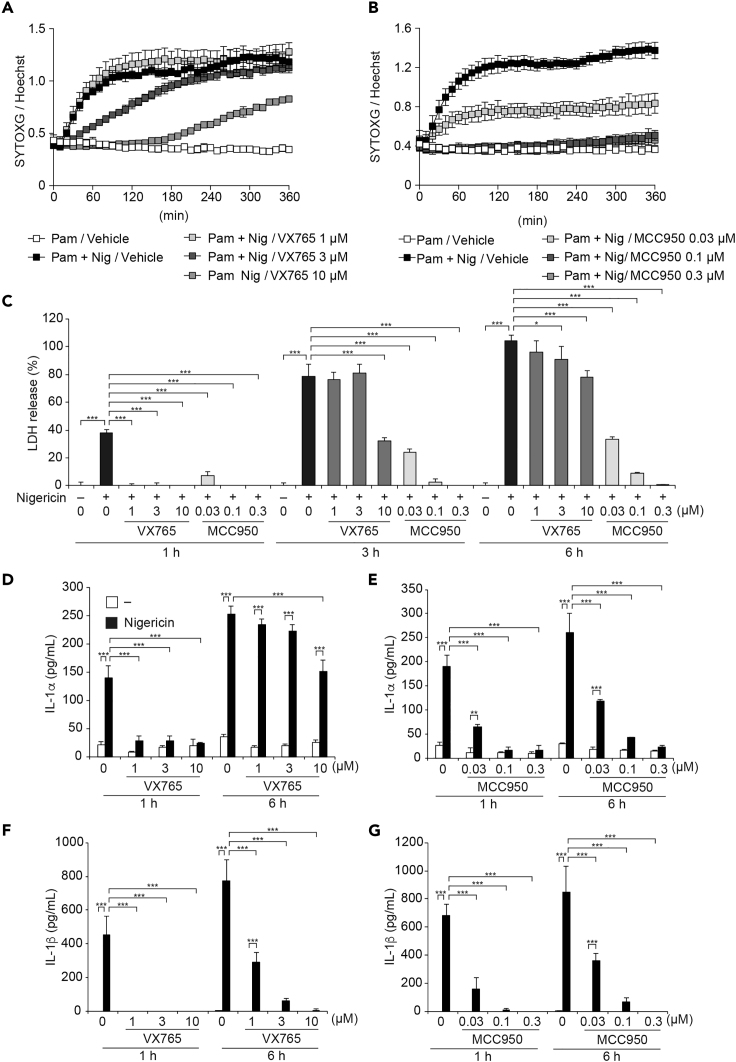
Pharmacological Inhibition of Casp1 Activation Dissociates IL-1β Release and Pyroptosis (A–G) Pam3CSK4-primed WT peritoneal macrophages were pretreated with VX-765 (1–10 μM) or MCC950 (0.03–0.3 μM) for 30 min and then treated with nigericin (5 μM). Relative fluorescence units of SYTOXG in (A)VX765-treated cells and (B) MCC950-treated cells were measured at 30-min intervals. (C) The levels of LDH in the supernatants were assessed. (D and E) The levels of IL-1α and (F and G) IL-1β in the supernatants were assessed by ELISA. (A–G) Data are shown as mean ± SD of triplicate of one experiment and representative of two independent experiments. ∗p < 0.05, ∗∗p < 0.01, ∗∗∗p < 0.001 as determined by two-way ANOVA with a post hoc test.

Inflammasome-targeting drugs have been developed over the past decade for application in inflammatory diseases (Coll et al., 2015, Lee et al., 2019). In particular, both NLRP3 and caspase-1 have been regarded as possible targets for inflammasome-targeting drugs. Therefore, we investigated the effects of NLRP3-targeting MCC950 and caspase-1-targeting VX765 on IL-1α/β release and pyroptosis. Consistent with the results in Casp1/11^−/−^ macrophages, VX765 dose-dependently delayed the onset of nigericin-induced cell death in Pam3CSK4-primed WT mouse peritoneal macrophages (Figure 7A). In contrast, MCC950 dose-dependently inhibited nigericin-induced cell death (Figure 7B). Although VX765-treatment completely inhibited LDH release at 1 h after nigericin treatment, VX765 failed to prevent delayed LDH release at 3–6 h after nigericin-treatment (Figure 7C). Moreover, substantial amounts of IL-1α release were detected in VX765-treated macrophages during nigericin-induced necrotic cell death (Figure 7D), whereas IL-1α release in MCC950-treated macrophages was inhibited, as well as LDH release (Figure 7E). Unlike IL-1α release, IL-1β release was inhibited in both VX765- and MCC950-treated macrophages (Figures 7F and 7G). To clarify the mechanisms of delayed necrotic cell death under caspase-1 inhibition, we assessed the processing of gasdermins. Reduced GSDMD processing and increased GSDME processing were detected in stimulated VX765-treated macrophages (Figure S7A), indicating that the nigericin-induced necrotic cell death in VX765-treated macrophages is pyroptosis. Furthermore, we confirmed that caspase-11 was not involved in delayed pyroptosis in VX765-treated macrophages using caspase-11-mutated macrophages (Figures S7B–S7D). These results suggest that pharmacological inhibition of caspase-1 dissociates IL-1α release and IL-1β release during pyroptosis.

## Discussion

The major findings of this study are as follows: (1) the activation of NLRP3 inflammasome induces necrotic cell death in the absence of caspase-1/11; (2) this caspase-1/11-independent necrotic cell death is mediated by ASC; (3) ASC initiates caspase-8-mediated GSDME-dependent pyroptosis and RIPK3-mediated necroptosis; (4) IL-1α, but not IL-1β, is released during Casp1/11-independent pyroptosis; (5) unprocessed pro-IL-1β is retained in pyroptotic cells; and (6) pharmacological inhibition of caspase-1 permits selective IL-1α release during pyroptosis. These results clearly suggest that activation of the NLRP3 inflammasome induces alternative pathways of necrotic cell death accompanied by IL-1α release under caspase-1 inhibition. To initiate these necrotic cell death programs, ASC plays an essential role by activating caspase-8.

A previous study has suggested that NLRP3 inflammasome activation drives GSDMD-independent pyroptosis with increased activation of caspase-8 (Schneider et al., 2017). However, the precise mechanisms regarding GSDMD-independent pyroptosis remain unclear. In the present study, we demonstrated that ASC-mediated activation of caspase-8 promotes the processing of GSDME to induce pyroptosis in *Casp1/11*-deficient macrophages. In contrast, recent studies have suggested that caspase-8 activation during *Yersinia* infection processes GSDMD to induce pyroptosis independent of canonical NLRP3 inflammasome activation (Orning et al., 2018, Sarhan et al., 2018). However, our results clearly demonstrated that NLRP3 inflammasome-mediated activation of caspase-8 failed to process GSDMD in *Casp1/11*-deficient macrophages. Instead, NLRP3 inflammasome promoted processing of GSDME as an alternative executor of pyroptosis in *Casp1/11*-deficient macrophages. Furthermore, the active caspase-8 induced by ectopic expression of ASC preferred to process GSDME rather than GSDMD in HeLa cells. Thus, we assume that GSDME, but not GSDMD, contributes to pyroptosis mediated by NLRP3 inflammasome under caspase-1 inhibition. On the other hand, GSDME processing was detected even in the absence of caspase-3, which is responsible for GSDME processing (Rogers et al., 2017, Wang et al., 2017). In addition, selective inhibition of caspase-8 preferentially prevented GSDME processing compared with caspase-3 inhibition. Although we did not find direct evidence that caspase-8 processes GSDME, we consider that caspase-8 is essential for GSDME-mediated pyroptosis induced by NLRP3 inflammasome under caspase-1 inhibition.

Other important issues are capability of inflammasome-mediated cell death induction and resultant cell death form in the absence of caspase-1. Presumably, they might be altered depending on the priming duration and cell types. Indeed, we found that short priming duration (4 h) decreased the capability of inflammasome-mediated necrotic cell death in Casp1/11-deficient macrophages. In this regard, a previous study has suggested that c-FLIP, which functions as a modulator of caspase-8, is upregulated by toll-like receptor-mediated signals and prevents ASC/caspase-8-mediated cell death (Van Opdenbosch et al., 2017). Although it is unclear whether long priming duration downregulates c-FLIP expression, c-FLIP could be a possible candidate for regulating capability of ASC/caspase-8-mediated cell death. Meanwhile, the form of caspase-8-mediated cell death could be affected by expression profiles of GSDME. We demonstrated that ectopic expression of GSDME altered ASC/caspase-8-mediated apoptosis to pyroptosis in HeLa cells. In contrast, the previous study has suggested that inflammasome-mediated caspase-8 activation induced apoptosis in *Casp1/11*-deficient bone marrow-derived dendritic cells (Antonopoulos et al., 2015). Further analyses are required to clarify the relationship between GSDME expression patterns and ASC/caspase-8-mediated cell death in various cell types. Notably, the processing of GSDME was induced by inflammasome activation even in caspase-1-expressing THP-1 cells, whereas nigericin-induced inflammasome activation failed to induce GSDME processing in WT murine peritoneal macrophages. The processed GSDME in caspase-1-expressing THP-1 cells is dispensable for pyroptosis because a similar LDH release was detected in control and *GSDME* KO THP-1 cells. Given that, however, the inflammasome-mediated processing of GSDME is determined by expression patterns of caspase-1 and caspase-8, the cells expressing low levels of caspase-1 and high levels of caspase-8 might employ GSDME for induction of pyroptosis.

Besides pyroptosis, our results suggest that necroptosis is involved in necrotic cell death in Z-VAD-treated *Casp1/11*-deficient macrophages during NLRP3 inflammasome activation because RIPK3 inhibitor significantly prevented nigericin-mediated necrotic cell death in them. We assume that NLRP3 inflammasome initiates necroptosis only when both caspase-1 and caspase-8 are inhibited because phosphorylation of RIPK3 and MLKL was induced only in the presence of Z-VAD. Thus, GSDME-mediated pyroptosis is a primary pathway for necrotic cell death induced by NLRP3 inflammasome under caspase-1 inhibition.

Another important finding in this study is the distinct mechanisms of IL-1α and IL-1β release during pyroptosis. Although IL-1β release was abrogated by the deficiency of caspase-1/11, a substantial amount of IL-1α was released during caspase-1/11-independent necrotic cell death including pyroptosis and necroptosis. Antonopoulos et al. previously reported that, in the absence of caspase-1, caspase-8 is alternatively activated and promotes the processing of IL-1β during NLRP3 inflammasome activation (Antonopoulos et al., 2015). However, our results demonstrated that caspase-8-mediated production of mature IL-1β was extremely limited; instead, partially processed (p28) IL-1β was detected. Moreover, pro-IL-1β was retained in pyroptotic cells after NLRP3 inflammasome activation in the absence of caspase-1. Although the mechanism by which unprocessed pro-IL-1β is retained in pyroptotic cells is unclear, the size of gasdermin pores may limit the release of high-molecular-weight complex containing pro-IL-1β. According to the predicted three-dimensional structure, the estimated size of pro-IL-1β is 6–7 nm (Figure S8); therefore, pro-IL-1β monomer is small enough to be released through GSDMD pores, which range from 13 to 22 nm (Aglietti et al., 2016, Mulvihill et al., 2018). Importantly, cleavage-defective mutants of pro-IL-1β were not released from pyroptotic cells, indicating that processing of IL-1β is required for its release during pyroptosis. In this regard, Monteleone et al. reported that processed mature IL-1β interacts with phosphatidylinositol-rich plasma membrane to promote efficient IL-1β release (Monteleone et al., 2018). This mechanism may also contribute to the selective release of mature IL-1β from pyroptotic cells. On the other hand, necrotic stimuli, such as ATP, endogenous crystals, or particulate matter, induce leakage of pro-IL-1β (Gross et al., 2012). However, the pathophysiological role of released pro-IL-1β has not been determined. Multiple studies suggest that a proteinase other than caspase-1, such as neutrophil proteinases, processes pro-IL-1β to initiate inflammatory responses (Mizushina et al., 2019, Netea et al., 2015, Sadatomo et al., 2017). Thus, leakage of pro-IL-1β and subsequent processing by other proteinases could enhance inflammatory responses. In contrast, retention of pro-IL-1β in pyroptotic cells might contribute to the control of excess inflammation by preventing proteinase-mediated pro-IL-1β processing in acute inflammation.

Recently, two studies investigated the form of cell death in the absence of GSDMD. The first study suggested that NLRP3 inflammasome activation induces secondary pyroptosis in the absence of GSDMD (Schneider et al., 2017). Although the mechanism of the induction of necrotic cell death is similar to that in our study, the phenotype associated with IL-1β release is different. Since caspase-1 is still active in GSDMD-independent secondary pyroptosis (Schneider et al., 2017), processed IL-1β is released in secondary pyroptosis. In contrast, the present study suggests that pyroptosis under caspase-1 inhibition is not accompanied by IL-1β release, a key feature of pyroptosis. Thus, we defined pyroptosis under caspase-1 inhibition as incomplete pyroptosis, which lacks IL-1β release but is accompanied by IL-1α release. The second study demonstrated that caspase-1 activation induces Bid/caspase-9-dependent apoptosis in the absence of GSDMD (Tsuchiya et al., 2019). In this regard, in the present study we showed that caspase-8 functions as an initiator of caspase in GSDME-dependent incomplete pyroptosis. Taken together, the features of GSDMD-independent secondary pyroptosis or apoptosis are distinct from those of incomplete pyroptosis under caspase-1 inhibition.

IL-1α release during incomplete pyroptosis was also observed under pharmacological inhibition of caspase-1. Treatment with VX-765 failed to inhibit IL-1α release and pyroptosis during NLRP3 inflammasome activation, whereas MCC950 completely inhibited NLRP3 inflammasome-mediated responses including IL-1α/β release and pyroptosis. Although both IL-1α and IL-1β target IL-1 receptor, these cytokines have been suggested to have distinct functions (Netea et al., 2015). In particular, a recent study found that IL-1α is involved in specific processes such as thrombopoiesis after platelet loss and wound healing (Burzynski et al., 2019). Thus, pharmacological inhibition of caspase-1 may provide a new therapeutic option by permitting selective IL-1α release in inflammatory responses.

Taken together, our findings demonstrate that NLRP3 inflammasome activation induces incomplete pyroptosis accompanied by IL-1α release under caspase-1 inhibition. We assume that regulation of this complex cell death mechanism will be valuable for appropriate therapy of inflammatory diseases.

### Limitations of the Study

In this study, we clarified that NLRP3 inflammasome activation induces caspase-1/11-independent necrotic cell death by activating several alternative pathways. However, this study had several limitations: (1) Although *in vitro* experiments clearly demonstrated that incomplete pyroptosis was induced by NLRP3 inflammasome activation under caspase-1 inhibition, the physiological significance of this cell death is still unclear. (2) With regard to the first issue, the processing of GSDME in an *in vivo* model was not determined in this study. (3) Caspase-8 presumably promotes the GSDME processing in the absence of caspase-3. However, we cannot exclude the possibility of GSDME processing by remaining caspase-3 because our *CASP1/CASP3* DKO cells were not cloned. At least, caspase-8 plays a pivotal role as an initiator of GSDME processing. (4) In the present study, inducers of incomplete pyroptosis are limited to nigericin and mutated NLRP3. On the other hand, lysosomal damaging stimuli such as cholesterol crystals and nanosilica particles promoted necrotic cell death independent of inflammasome. Further investigations are needed to clarify the role of incomplete pyroptosis in inflammatory responses.

## Methods

All methods can be found in the accompanying Transparent Methods supplemental file.

## Supplemental Information

Document S1. Transparent Methods and Figures S1–S8
