# Supplementary material for: GSDME-Dependent Incomplete Pyroptosis Permits Selective IL-1α Release under Caspase-1 Inhibition
Source: iScience. 2020 Apr 18;23(5):101070. doi: 10.1016/j.isci.2020.101070 (PMC7200307; doi:10.1016/j.isci.2020.101070)
Supplement: Document S1. Transparent Methods and Figures S1–S8 [file mmc1.pdf]

iScience, Volume *23*

Supplemental Information

GSDME-Dependent Incomplete Pyroptosis Permits Selective IL-1α Release under Caspase-1 Inhibition

Emi Aizawa, Tadayoshi Karasawa, Sachiko Watanabe, Takanori Komada, Hiroaki Kimura, Ryo Kamata, Homare Ito, Erika Hishida, Naoya Yamada, Tadashi Kasahara, Yoshiyuki Mori, and Masafumi Takahashi

Supplemental Information

Transparent Methods

CONTACT FOR REAGENT AND RESOUCE SHARING

Requests for resources, reagents, and further information should be directed to, and fulfilled by Tadayoshi Karasawa (tdys.karasawa@jichi.ac.jp)

METHODS DETAILS

Animals

C57BL/6J mice were purchased from SLC Inc. (Shizuoka, Japan). *Nlrp3*$^{-/-}$, *Casp1/11*$^{-/-}$, and *Asc*$^{-/-}$ mice were provided by Dr. Vishva M. Dixit, Dr. Hiroko Tsusui, and Dr. Shun'ichiro Taniguchi, respectively (Kuida et al., 1995; Mariathasan et al., 2006; Yamamoto et al., 2004). *Asc*$^{-/-}$ *Casp1/11*$^{-/-}$ mice were developed by crossing *Casp1/11*$^{-/-}$ mice with *Asc*$^{-/-}$ mice. *Casp1*$^{flox/flox}$*Casp11*$^{-/-}$ mice were generated from 129 ES cells by PhoenixBio (Hiroshima, Japan) and backcrossed with C57BL/6J for 12 generations. All genetically modified mice used in this study had a C57BL/6J background. Only male mice were used to exclude influences of the female hormonal cycle. For isolation of thioglycollate-elicited macrophages, mice were injected intraperitoneally with 1 mL of 4% Brewer thioglycollate medium (211716, Becton Dickinson, Franklin Lakes, NJ), and peritoneal cells were collected 3 days after injection. The isolated cells were pooled per genotype and seeded at 1 x 10^6 cells/mL in 10% fetal calf serum (FCS)/RPMI 1640 medium. After 3 h, non-adherent cells were washed out by phosphate-buffered saline (PBS) and adherent cells were used as peritoneal macrophages. For preparation of bone marrow-derived macrophages (BMDMs), bone marrow cells were isolated from the femurs and tibias of the mice and cultured in RPMI 1640 medium supplemented with 10% FCS and 15% conditioned medium of L929 cells (ATCC, Rockville, MD) for 7 days at density of 2 x 10^6 cells/mL. All animal experiments were approved by the Use and Care of Experimental Animals Committee of the Jichi

Medical University Guide for Laboratory Animals, and carried out in accordance with the Jichi Medical University guidelines.

Cell lines

LentiX293T (Takara Bio, Shiga, Japan) cells were cultured in Dulbecco's modified Eagle's medium (DMEM; Wako, Osaka, Japan) supplemented with 10% FCS, 1 mM sodium pyruvate, and antibiotics. THP-1 cells were cultured in RPMI1640 (Sigma, St Louis, MO, USA) supplemented with 10% FCS and antibiotics. THP-1 cells were differentiated with 200 nM PMA (Wako) for 24 or 48 h. HeLa cells were cultured in DMEM supplemented with 10% FCS and antibiotics.

Plasmids

The polymerase chain reaction (PCR)-generated cDNAs encoding hKO1, human ASC, CASP1, GSDMD, GSDME, and NLRP3 were subcloned into pCDNA3.1 vector (Thermo Fisher Scientific, Waltham, MA, USA) with or without tags (N-terminal 3 x Flag or N-terminal Myc). The lentiviral vector plasmids encoding human IL1B were developed as previously described (Mizushina et al., 2019). The mutated NLRP3D303N and IL1BD27A were generated using the PrimeSTAR Mutagenesis Basal kit (Takara Bio) with the following primers: (NLRP3D303N forward, 5′-GGCTTCAATGAGCTGCAAGGTGCCTTTGACGAG-3′; NLRP3D303N reverse, 5′-CAGCTCATTGAAGCCGTCCATGAGGAAGAGGAT-3′; IL1BD27A forward, 5′-GAAGCTGCTGGCCCTAAACAGATGAAG -3′; reverse, 5′-AGGGCCAGCAGCTTCAAAGAACAAGTC -3′). To produce doxycycline-inducible expression vector, NLRP3D303N was inserted into pENTR4 and then transferred into CS-IV -TRE-RfA-CMV-KT (kindly provided by Dr. Hiroyuki Miyoshi, RDB12876, RIKEN BRC, Tsukuba, Japan) with LR clonase (Thermo Fisher Scientific).

Lentiviral preparation

LentiX293T cells were co-transfected together with LentiCRISPRv2, pLP1, pLP2, and pVSVG using PEI MAX (Polysciences, Warrington, PA, USA) to prepare the lentiviral vectors. Culture media containing the lentiviral vectors were collected 3 days after transfection. The collected media were filtered with a 0.45-μm filter and ultracentrifuged at 21,000 rpm using a SW55 Ti rotor (Beckman Coulter, Brea, CA, USA), and the pellets were resuspended in PBS containing 5% FCS. The lentivirus titer was measured using a Lentivirus qPCR Titer kit (Applied Biological Materials, Richmond, BC, Canada).

CRISPR/Cas9-mediated genome editing in THP-1 cells

ASC, *CASP1*, *CASP3*, *CASP8*, *GSDMD*, and *GSDME* genes were mutated by CRISPR/Cas9 in THP-1 cells. The sgRNA targeting each gene was designed with CRISPR direct (http://crispr.dbcls.jp) and subcloned into LentiCRISPRv2, which was a gift from Feng Zhang (Addgene plasmid #52961; http://n2t.net/addgene: 52961; RRID: Addgene_52961), or its progeny harboring blasticidin-resistant gene. For lentiviral transduction, THP-1 cells were incubated with lentiviral vectors for 16 h in the presence of 8 μg/mL polybrene (Sigma). The transduced cells were selected by incubating them with 2 μg/mL puromycin (Sigma) for at least 3 days. To produce cells with multiple mutation, the transduced cells were further selected by blasticidin (Wako).

Development of THP-1NLRP3D303N cells

THP-1 cells were transduced with CSIV-TRE-NLRP3D303N-CMV-KT lentiviral vector at MOI 300, and transduced cells were then subjected to limited dilution cloning. To produce *CASP1*KO or *ASC* KO *NLRP3D303N*-THP-1 cells, cells were transduced with LentiCRISPRv2 vector as described above and further separated by limited dilution.

Transient transfection in HeLa cells

Transient transfection was performed using Lipofectamine 2000 (Thermo Scientific). HeLa cells were seeded at 1×10^5 cells/mL and cultured for 24 h. HeLa cells were transfected with plasmids encoding NLRP3D303N, ASC, Caspase-1, GSDMD and GSDME for 6 h. After transfection, medium was changed from DMEM to Opti-MEM (Thermo Scientific).

IL-1 secretion assay

Primary peritoneal macrophages isolated from WT, *Casp1/11*$^{-/-}$, and *Asc*$^{-/-}$ *Casp1/11*$^{-/-}$ mice were seeded at 1.25×10^5 cells/well into 96 well plates. *NLRP3D303N*-THP-1 cells were seeded at 5×10^4 cells/well into 96 well plates. After indicated treatments, culture supernatants were collected and the IL-1α and IL-1β levels were measured by enzyme-linked immunosorbent assay (ELISA) using a commercial kit (R&D Systems, Minneapolis, MN, USA). The supernatants were precipitated with ice-cold acetone and resolved in 1× Laemmli buffer for western blot analysis.

LDH release assay

Primary peritoneal macrophages isolated from WT, *Casp1/11*$^{-/-}$, and *Asc*$^{-/-}$ *Casp1/11*$^{-/-}$ mice were seeded at 1.25×10^5 cells/well into 96 well plates. *NLRP3D303N*-THP-1 cells and THP-1 cells were seeded at 5×10^4 cells/well into 96 well plates. After indicated treatments, culture supernatants were collected. Hela cells were seeded at 1.25×10^4 cells/well into 96 well plates one day before the transfection. Culture supernatants were collected at 24 h after transfection. LDH released from cells was measured by a Cytotoxicity Detection kit (Roche, Mannheim, Germany) according to the manufacturer's instructions. To determine the total cellular LDH activity, cells were lysed with 2% TritonX100. L-Lactate Dehydrogenase from rabbit muscle (Roche) was used as a standard.

SYTOX Green assay

Cells were incubated with 1 μg/mL Hoechst33342 (Dojindo, Kumamoto, Japan) for 20 min, and then cultured in the presence of 100 nM SYTOX Green (SYTOXG; Thermo Fisher Scientific) for 30 min. After labeling, cells were stimulated with reagents. Fluorescence intensity was measured by using a multimode microplate reader (Spark; TECAN, Switzerland).

Live cell imaging

Peritoneal macrophages were seeded as at 2.5 $\times 10^5$ cells on an 8-well coverglass chamber (IWAKI, Shizuoka, Japan) and primed with Pam3CSK4 for 18 h. After labeling with 1μg/mL Hoechst33342 for 20 min, cells were cultured in medium containing 100 nM SYTOXG. After images were obtained at 0 h, cells were stimulated with reagents and images were captured at the indicated time points using confocal microscopy (FLUOVIEW FV10i; Olympus, Tokyo, Japan). For imaging of HeLa cells, cells were seeded at 2.5×10^4 cells on an 8-well coverglass chamber and cultured for 24 h. Cells were transiently transfected with plasmids for 6 h, and then the medium was changed to Opti-MEM and the cells were cultured for 24 h. Before the examination, cells were labeled with SYTOXG and Hoechst33342. To evaluate the nuclear size of dead cells, images of SYTOXG staining were analyzed by Image J (1.52q; National Institutes of Health, Bethesda, MD, USA).

Crosslinking assay

Cells were lysed in cross-linking buffer (20 mM phosphate buffer, pH 8.0, 150 mM NaCl, and 1% NP-40) for the cross-linking assay. After centrifugation, the supernatants were placed on ice with 2mM BS3 (Thermo Fisher Scientific) for 2 h, and the reaction was terminated by adding an excess amount of glycine.

Isolation of Triton X100-insoluble fraction

Cells were lysed in 0.5% Triton X-100 buffer (20 mM Tris HCl, pH7.4, 10 mM KCl, 1.5 mM MgCl2, 1 mM EDTA, 1 mM EGTA, 320 mM sucrose, and 0.5% Triton X-100) for 20

min, and lysates were centrifuged at 5,000xg for 10 min. The supernatants were collected as soluble fraction. The insoluble pellets were resolved in 1× Laemmli buffer for western blot analysis.

Western blot analysis

Samples were separated by sodium dodecyl sulfate-polyacrylamide electrophoresis (SDS-PAGE) and transferred to PVDF membranes. After blocking with Blocking One (NACALAI TESQUE, Kyoto, Japan) for 30 min, the membranes were incubated overnight at 4ºC with the following primary antibodies: anti-ASC (AG-25B-0006; Adipogen, Farmingdale, NY, USA), anti-β actin, (A5441; Sigma), anti-caspase-1(AG-20B-0042-C100; Adipogen), anti-caspase-1(#3866; Cell Signaling Technology, Danvers, MA), anti-caspase-3 (#9665; Cell Signaling Technology), anti-cleaved caspase-8 (#9496; Cell Signaling Technology), anti-caspase-8 (#9746; Cell Signaling Technology), anti-cleaved caspase-8 (#8952; Cell Signaling Technology), anti-caspase-11 (#14340; Cell Signaling Technology), anti-GSDMD (#50928; Cell Signaling Technology), anti-GSDMD (G7422; Sigma), anti-GSDMD (ab209845; Abcam, Cambridge, UK), anti-GSDME (ab215191; Abcam), anti-IL-1β (sc-7884; Santa Cruz Biotechnology, Dallas, TX, USA), anti-IL-1α (AF-400-SP; R&D Systems), anti-IL-1β (AF-401NA; R&D Systems), and anti-NLRP3 (AG-20B-0014; Adipogen), anti-MLKL (MABC604; Sigma), anti-phospho-MLKL (ab196436; Abcam), anti-RIP3 (#95702; Cell Signaling Technology) , anti-phospho-RIP3 (#57220; Cell Signaling Technology) antibodies. As secondary antibodies, HRP-goat anti-mouse Superclonal IgG (Thermo Fisher Scientific), HRP-goat anti-rabbit IgG (Cell Signaling Technology), HRP-rabbit anti-goat IgG (Thermo Fisher Scientific) were incubated with membrane for 1 h. After washing with TBS-Tween, immunoreactive bands were visualized by Western Blot Quant HRP substrate (TAKARA Bio) or Western BLoT Ultra Sensitive HRP substrate (TAKARA Bio).

Statistical analysis

Data are expressed as mean ± standard deviation (SD). Differences between two groups were determined by Mann-Whitney's U-test. Differences between multiple group means were determined by two-way analysis of variance (ANOVA) combined with the Tukey's post hoc test. Differences between multiple groups with repeated measurements were determined by repeated one-way ANOVA or repeated two-way ANOVA combined with the post hoc test. All analyses were performed using GraphPad Prism 6 software (Graph Pad Software, La Jolla, CA, USA). A p-value of < 0.05 was considered statistically significant.

Prediction of IL-1β diameter

The three-dimensional structure of IL-1β pro domain (1-116) was predicted by QUARK (University of Michigan, Ann Arbor, MI, USA). The obtained structures and mature IL-1β (PDB code 1I1B) were further analyzed by CRYSOL (Version 2.8.2; European Molecular Biology Laboratory, Hamburg, Germany).

Supplemental References

Kuida, K., Lippke, J.A., Ku, G., Harding, M.W., Livingston, D.J., Su, M.S., and Flavell, R.A. (1995). Altered cytokine export and apoptosis in mice deficient in interleukin-1 β converting enzyme. Science *267*, 2000-2003.

Mariathasan, S., Weiss, D.S., Newton, K., McBride, J., O'Rourke, K., Roose-Girma, M., Lee, W.P., Weinrauch, Y., Monack, D.M., and Dixit, V.M. (2006). Cryopyrin activates the inflammasome in response to toxins and ATP. Nature *440*, 228-232.

Mizushina, Y., Karasawa, T., Aizawa, K., Kimura, H., Watanabe, S., Kamata, R., Komada, T., Mato, N., Kasahara, T., Koyama, S., *et al.* (2019). Inflammasome-Independent and Atypical Processing of IL-1β Contributes to Acid Aspiration-Induced Acute Lung Injury. Journal of immunology *203*, 236-246.

Yamamoto, M., Yaginuma, K., Tsutsui, H., Sagara, J., Guan, X., Seki, E., Yasuda, K., Yamamoto, M., Akira, S., Nakanishi, K., *et al.* (2004). ASC is essential for LPS-induced

activation of procaspase-1 independently of TLR-associated signal adaptor molecules. Genes to cells : devoted to molecular & cellular mechanisms *9*, 1055-1067.

KEY RESOURCES TABLE

REAGENT or RESOURCE	SOURCE	IDENTIFIER
Antibodies		
Rabbit monoclonal anti-caspase-1 (D7F10)	Cell Signaling Technology	#3866
Rabbit monoclonal anti-caspase-3(8G10)	Cell Signaling Technology	#9665
Rabbit monoclonal anti-cleaved caspase-8	Cell Signaling Technology	#9496
Rabbit monoclonal anti-cleaved caspase-8	Cell Signaling Technology	#8592
Mouse monoclonal anti-Caspase-8	Cell Signaling Technology	#9746
Rat monoclonal anti-Caspase-11 (17D9)	Cell Signaling Technology	#14340
Rabbit polyclonal anti-GSDMD	Cell Signaling Technology	#50928
Rabbit monoclonal anti-RIP3	Cell Signaling Technology	#95702
Rabbit polyclonal anti-phospho-RIP3	Cell Signaling Technology	#57220
Rabbit polyclonal anti-GSDMD	Sigma-Aldrich	G7422
Rabbit monoclonal anti-GSDMD	Abcam	ab209845
Rabbit monoclonal anti-GSDME	Abcam	ab215191
Rabbit monoclonal anti-phospho-MLKL	Abcam	Ab196436
Mouse monoclonal anti-NLRP3	Adipogen	AG-20B-0014
Rabbit polyclonal anti-ASC	Adipogen	AG-25B-0006
Mouse monoclonal anti-Caspase-1 (p20)	Adipogen	AG-20B-0042-C100

Rabbit polyclonal anti-IL-1β	Santa Cruz	sc-7884
Goat polyclonal anti-IL-1β	R&D	AF-401-NA
Goat polyclonal anti-IL-1α	R&D	AF-400-SP
Mouse monoclonal anti-β-actin	Sigma-Aldrich	A5441
Rat monoclonal anti-MLKL	Sigma-Aldrich	MABC604
Chemicals, Peptides, and Recombinant Proteins		
MCC950	AdipoGen	AG-CR1-3615-M005
Nigericin	Invivo Gen	tlrl-nig
Pam3CSK4	Invivo Gen	tlrl-pms
Z-DEVD-FMK	MBL	4800-510
Z-IETD-FMK	MBL	4805-510
Z-VAD-FMK	MBL	4800-520
PEI MAX	Polysciences	24765-1
VX765	Selleck	S2228
GSK'872	Selleck	S8465
Puromycin	Sigma-Aldrich	P8833
Lipofectamine 2000	Thermo Fisher Scientific	11668019
SYTOX Green	Thermo Fisher Scientific	S7020
Blasticidin S Hydrochloride	Wako	029-18701
Phorbol 12-Myristate 13-Acetate	Wako	162-23591
Doxycycline Hydrochloride n-Hydrate	Wako	049-31121
Critical Commercial Assays		
Lentiviral qPCR Titration Kit	Applied Biological Materials	#LV900
Cytotoxicity Detection Kit (LDH)	Roche	11644793001
Human IL-1 beta/IL-1F2 DuoSet ELISA	R＆D Systems	DY201
Mouse IL-1 beta/IL-1F2 DuoSet ELISA	R＆D Systems	DY401
Mouse IL-1 alpha/IL-1F1 DuoSet ELISA	R＆D Systems	DY400
Experimental Models: Cell Lines		

LentiX293T	Takara Bio	Z2180N
Hela	ATCC	CCL-2
Hela *CASP3* KO	This manuscript	N/A
THP-1	ATCC	TIB-202
THP-1 *CASP1* KO	This manuscript	N/A
THP-1 *ASC* KO	This manuscript	N/A
THP-1 *CASP1* / *CASP8* DKO	This manuscript	N/A
THP-1 *GSDMD* KO	This manuscript	N/A
THP-1 *GSDME* KO	This manuscript	N/A
THP-1 *GSDMD* / *GSDME* DKO	This manuscript	N/A
THP-1 *NLRP3D303N*	This manuscript	N/A
THP-1 *NLRP3D303N CASP1* KO	This manuscript	N/A
THP-1 *NLRP3D303N ASC* KO	This manuscript	N/A
THP-1 *NLRP3D303N* / *KO1*	This manuscript	N/A
THP-1 *NLRP3D303N* / *hIL1B*	This manuscript	N/A
THP-1 *NLRP3D303N* / *hIL1BD27A*	This manuscript	N/A
THP-1 *NLRP3D303N* / *hIL1BD116I*	This manuscript	N/A
THP-1 *NLRP3D303N* / *hIL1BD27A / D116I*	This manuscript	N/A
Experimental Models: Organisms/Strains		
Mouse: C57BL/6J	SLC	N/A
Mouse: $Casp1^{-/-}Casp11^{-/-}$	Kuida et al., 1995	N/A
Mouse: $Asc^{-/-}$	Yamamoto et al., 2004	N/A
Mouse: $Asc^{-/-}Casp1^{-/-}Casp11^{-/-}$	This manuscript	N/A
Mouse: $Nlrp3^{-/-}$	Mariathasan et al., 2006	N/A
Mouse: $Casp1^{flox/flox}$ $Casp11^{-/-}$	This manuscript	N/A
Oligonucleotides		
sgRNA sequence for GFP– GAGCTGGACGGCGACGTAAA	This manuscript	N/A
sgRNA sequence for CASP1– AAGCTGTTTATCCGTTCCAT	This manuscript	N/A

sgRNA sequence for ASC–<br>TCTTGAGCTCCTCGGCGGTC	This manuscript	N/A
sgRNA sequence for GSDMD–<br>TGAGCGGGTAGTCCGGAGAG	This manuscript	N/A
sgRNA sequence for CASP8#1–<br>GCCTGGACTACATTCCGCAA	This manuscript	N/A
sgRNA sequence for CASP8#2–<br>AACATCAAGGCATCCTTGAT	This manuscript	N/A
sgRNA sequence for GSDME#1–<br>TCTTCTGTGTCAAAACGCAC	This manuscript	N/A
sgRNA sequence for GSDME#2–<br>GAGAAGTGTGGTGGCATCGT	This manuscript	N/A
sgRNA sequence for CASP3#1–<br>CATACATGGAAGCGAATCAA	This manuscript	N/A
sgRNA sequence for CASP3#2–<br>ATTATACATAAACCCATCTC	This manuscript	N/A
Mutation PCR primer for NLRP3D303N Forward–<br>GGCTTCAATGAGCTGCAAGGTGCCTTTGACGAG	This manuscript	N/A
Mutation PCR primer for NLRP3D303N Reverse–<br>CAGCTCATTGAAGCCGTCCATGAGGAAGAGGAT	This manuscript	N/A
Mutation PCR primer for IL1BD27A Forward–<br>GAAGCTGCTGGCCCTAAACAGATGAAG	This manuscript	N/A
Mutation PCR primer for IL1BD27A Reverse–<br>AGGGCCAGCAGCTTCAAAGAACAAGTC	This manuscript	N/A
Recombinant DNA		
pcDNA3.1KO1	This manuscript	N/A
pcDNA3.1 human ASC	This manuscript	N/A
pcDNA3.1 Myc(N) human CASP1	This manuscript	N/A
pcDNA3.1 human GSDMD	This manuscript	N/A
pcDNA3.1 human GSDME	This manuscript	N/A
pcDNA3.1 3xFlag(N) human GSDMD	This manuscript	N/A
pcDNA3.1 3xFlag(N) human GSDME	This manuscript	N/A

CSIV-TRE-RfA-CMV-KT	RIKEN BRC	N/A
CSIV-TRE-NLRP3 D303N-CMV-KT	This manuscript	RDB12876
CSCA-MCS	RIKEN BRC	RDB05963
CSCAKO1	Mizushina et al., 2019	N/A
CSCA human IL1BFlagHis	Mizushina et al., 2019	N/A
CSCA human IL1BD27AFlagHis	This manuscript	N/A
CSCA human IL1BD116IFlagHIs	Mizushina et al., 2019	N/A
CSCA human IL1BD27A/D116IFlagHis	This manuscript	N/A
LentiCRISPRv2	Addgene	#52961
LentiCRISPRv2 sgGFP	This manuscript	N/A
LentiCRISPRv2 sgCASP1	This manuscript	N/A
LentiCRISPRv2 sgASC	This manuscript	N/A
LentiCRISPRv2 sgGSDMD	This manuscript	N/A
LentiCRISPRv2 sgCASP3	This manuscript	N/A
LentiCRISPRBsd	This manuscript	N/A
LentiCRISPRBsd GFP	This manuscript	N/A
LentiCRISPRBsd CASP8	This manuscript	N/A
LentiCRISPRBsd CASP3	This manuscript	N/A
LentiCRISPRBsd GSDME	This manuscript	N/A
Software and Algorithms		
GraphPad Prism 6	Graph Pad Software	
Image J	National Institute of Health	1.52q
CRYSOL	European Molecular Biology Laboratory	Version 2.8.2
QUARK	University of Michigan	

Figure S1. Nigericin-induced necrotic cell death in Casp1/11$^{-/-}$ macrophages, Related to Figure 1

(A and B) Primary peritoneal macrophages isolated from WT, *Nlrp3*$^{-/-}$, or *Casp1/11*$^{-/-}$ mice were rested or primed with Pam3CSK4 (100 ng/mL) for 18 h, and then treated with nigericin (1.5 μM) for 1 h or 6 h. (A) The levels of LDH in the supernatants from WT and *Nlrp3*$^{-/-}$ macrophages were assessed. (B) The levels of LDH in the supernatants from WT and *Casp1/11*$^{-/-}$ macrophages were assessed. (C–E) Primed WT and *Casp1/11*$^{-/-}$ macrophages were labelled with Hoechst33342, and then treated with nigericin in the presence of SYTOX Green. (C and D) Images were visualized by confocal microscopy. (E) Nuclear sizes of dead cells were quantified (WT: n = 161, *Casp1/11*$^{-/-}$: n = 94). (F and G) Control and *CASP1* KO THP-1 cells were differentiated with PMA for 48 h, and then treated with nigericin (5 μM) in the presence of SYTOXG. (F) Images were visualized by confocal microscopy and (G) nuclear sizes of dead cells were quantified (Control: n=70, *CASP1* KO: n= 35). Data are shown as (A and B) mean ± SD of triplicate of one experiment or (E and G) box plot with medians indicated as horizontal bars within boxes. (C–E) Data are representative of two independent experiment. $^{**}P < 0.01$, $^{***}P < 0.005$ as determined by (A and B) two-way ANOVA with a post hoc test or (E and G) Mann-Whitney U test.

Figure S2. Caspase-1-independent necrotic cell death induced by inflammasome activation, Related to Figure 1

(A–C) Primary peritoneal macrophages isolated from WT, *Casp1/11*$^{-/-}$, and *Asc*$^{-/-}$ *Casp1/11*$^{-/-}$ mice were rested or primed with Pam3CSK4 (100 ng/mL) for 4 h and then treated with nigericin (5 μM) for 1 h, 3 h, or 6 h. (A) The levels of LDH in the supernatants were assessed. (B) The levels of IL-1β and (C) IL-1α in the supernatants were assessed by ELISA. (D) WT, *Casp1/11*$^{-/-}$, and *Asc*$^{-/-}$ *Casp1/11*$^{-/-}$ macrophages were primed with Pam3CSK4 (100 ng/mL) for 4 h and labelled with Hoechst33342. After nigericin stimulation, relative fluorescence units of SYTOXG were measured at 10-min intervals. (E) Primary peritoneal macrophages isolated from *Casp1/11*$^{-/-}$ mice were rested or primed with Pam3CSK4 (100 ng/mL) for 4 h or 18 h and then treated with

nigericin (5 μM) for 3 h or 6 h. The levels of LDH in the supernatants were assessed. (F) WT, *Casp1/11*$^{-/-}$, and *Asc*$^{-/-}$ *Casp1/11*$^{-/-}$ bone marrow-derived macrophages were primed with Pam3CSK4 (100 ng/mL) for 18 h, and then treated with nigericin (5 μM) for 1 h, 3 h, or 6 h. The levels of LDH in the supernatants were assessed. (G and H) Control, *ASC* KO, and *CASP1* KO THP-1 cells were differentiated with PMA for 48 h, and then treated with (G) Nigericin (5 μM) for 24 h or (H) Nanosilica particles (30 nm, 100 μg/mL), Cholesterol crystals (CH-C; 100 μg/mL), and Palmitic acid crystals (PA-C; 100 μg/mL) for 24 h. The levels of LDH in the supernatants were assessed. (A–G) Data are shown as mean ± SD of triplicate of one experiment. (A–G) Data are representative of two independent experiments. *$P < 0.05$, ***$P < 0.005$ as determined by two-way ANOVA with a post hoc test.

Figure S3. Development of THP-1 cells expressing NLRP3D303N mutants, Related to Figure 2

(A) Schematic model of lentiviral vector encoding NLRP3D303N mutant under the TET-ON promoter. The vector encodes hKO1 under the CMV promoter. (B–D) THP1 *NLRP3 D303N* cells were differentiated with PMA for 24 h and treated with doxycycline (DOX; 10, 100, 1000 ng/mL) for the indicated period. (B) Lysates and supernatants were analyzed by western blot after 6 h of DOX treatment. (C) LDH release in supernatants was measured. (D) The levels of IL-1β in supernatants were measured by ELISA. (E) Cells were stained with 7-AAD and FITC Annexin V and analyzed by flow cytometry. (F) THP-1 *NLRP3D303N* cells transduced with control lentiviral vectors were differentiated with PMA for 48 h and treated with DOX (1 μg/mL) in the presence of SYTOX Green. Images were visualized by confocal microscopy. (G) Control and *CASP1*KO THP1 *NLRP3 D303N* cells were differentiated with PMA for 48 h, and then treated with DOX (1 μg/mL) in the presence of SYTOXG. Images were visualized by confocal microscopy and nuclear sizes of dead cells were quantified (Control: n=128, *CASP1* KO: n= 52). Data are shown as (C and D) mean ± SD of triplicate of one experiment or (G) box plot with medians indicated as horizontal bars within boxes. (B–G) Data are representative of two

independent experiments. **$P < 0.01$, ***$P < 0.005$ as determined by (C and D) two-way ANOVA with a post hoc test or (G) Mann-Whitney U test.

Figure S4. Effect of caspase inhibition on caspase-1-independent necrotic cell death, Related to Figure 3

(A and B) *CASP1*KO THP1 *NLRP3 D303N* cells were differentiated with PMA for 48 h, and then treated with DOX (1 μg/mL) in the presence of Z-VAD (20 μM). (A) LDH release in supernatant was assessed. (B) Cells were stimulated in the presence of SYTOXG. Relative fluorescence units of SYTOXG were measured. (C) *CASP1*KO THP1 cells were pretreated with Z-VAD, Z-DEVD or Z-IETD (20 μM each) for 30 min, and treated with nigericin (5 μM) for 8 h. LDH release in supernatant was measured. (D) Primed Casp1/11$^{-/-}$ macrophages were labelled with Hoechst33342, and then treated with Z-VAD and nigericin in the presence of SYTOX Green. Images were visualized by confocal microscopy. Data are shown as mean ± SD of triplicate (A and C) or pentaplicate (B) of one experiment. (A–D) Data are representative of two independent experiments. *$P < 0.05$, ***$P < 0.005$ as determined by two-way ANOVA with a post hoc test.

Figure S5. NLRP3 inflammasome-mediated signaling independent of caspase-1, Related to Figure 4

(A) Primary peritoneal macrophages isolated from WT, *Casp1/11*$^{-/-}$ mice were rested or primed with Pam3CSK4 (100 ng/mL) for 18 h, and then treated with nigericin (5 μM) for 1 h, 3 h, or 6 h. Lysates and supernatants were collected at indicated time points and analyzed by western blot. (B) Alignment of amino acid sequences of human IL-1β and mouse IL-1β. Cleavage sites (ASP27 and ASP116) are indicated in red. (C and D) THP1 *NLRP3 D303N* /*hIL1B* cells were differentiated with PMA for 48 h and treated with DOX (1 μg/mL) for 6 h. (C) LDH release in supernatant was assessed. (D) Supernatants were analyzed by western blot. (E and F) Pam3CSK4-primed *Casp1/11*$^{-/-}$ macrophages were pretreated with Z-VAD (20 μM), and then

treated with nigericin (5 μM) for 6 h. (E) Separated soluble and insoluble fractions were analyzed by western blot. (F) Lysates were analyzed by western blot. (C) Data are shown as mean ± SD of triplicate of one experiment. (C–F) Data are representative of two independent experiments.

Figure S6. Effects of ASC-mediated caspase-8 activation on GSDME processing, Related to Figure 5 and Figure 6

(A) Lysates from control, *CASP1* KO, and *CASP1* and *CASP8* double-KO THP1 cells were analyzed by western blot. (B) Lysates from Hela cells and differentiated THP1 cells were analyzed by western blot (C) HeLa cells were transiently transfected with the indicated plasmids. After 24 h, cell lysates were analyzed by western blot. (D) Lysates from control and *CASP3* KO HeLa cells were analyzed by western blot. (E) Control, *CASP1* KO, and *CASP1* and *CASP3* double-KO THP1 cells were differentiated with PMA for 48 h. Lysates were analyzed by western blot. (F and G) *CASP1* KO, and *CASP1* and *CASP3* double-KO THP1 cells were differentiated with PMA for 48 h and then treated with nigericin (5μM) for 8 h. (F) Relative fluorescence units of SYTOXG were measured at 30-min intervals. (G) LDH release in supernatant was assessed. (H) Lysates from control, *GSDME* KO, *GSDMD* KO, *GSDMD* and *GSDME* KO were analyzed by western blot. (F and G) Data are shown as mean ± SD of triplicate of one experiment. (A–G) Data are representative of two independent experiments. $*P < 0.05$, $***P < 0.005$ as determined by two-way ANOVA with a post hoc test.

Figure S7. Pharmacological inhibition of caspase-1 in peritoneal macrophages, Related to Figure 7

(A) Primary peritoneal macrophages isolated from WT mice were pretreated with VX-765 (3 or 10 μM) or MCC950 (0.1 or 0.3 μM) for 30 min, and then treated with nigericin (5 μM). After 3 h, lysates and supernatants were analyzed by western blot. (B–D) Primary peritoneal macrophages isolated from WT mice and *Casp1*$^{flox/flox}$*Casp11*$^{-/-}$ mice were pretreated with VX-765

(1–10 μM) for 30 min, and then treated with nigericin. After 3 h, lysates and supernatants were analyzed by western blot. (C) Cells were stimulated in the presence of SYTOXG. Relative fluorescence units of SYTOXG were measured. (D) LDH release in supernatant was assessed. Data are shown as mean ± SD of triplicate (D) or pentaplicate (C) of one experiment. (C and D) Data are representative of two independent experiments. $^{**}P < 0.01$, $^{***}P < 0.005$ as determined by two-way ANOVA with a post hoc test.

Figure S8. Prediction of three-dimensional structure of IL-1β prodomain, Related to Figure 4

(A) The three-dimensional structure of IL-1β prodomain (1-116) was predicted by QUARK. The predicted structures of IL-1β prodomain and mature IL-1β were further analyzed by CRYSOL to calculate the diameter.

Figure S1

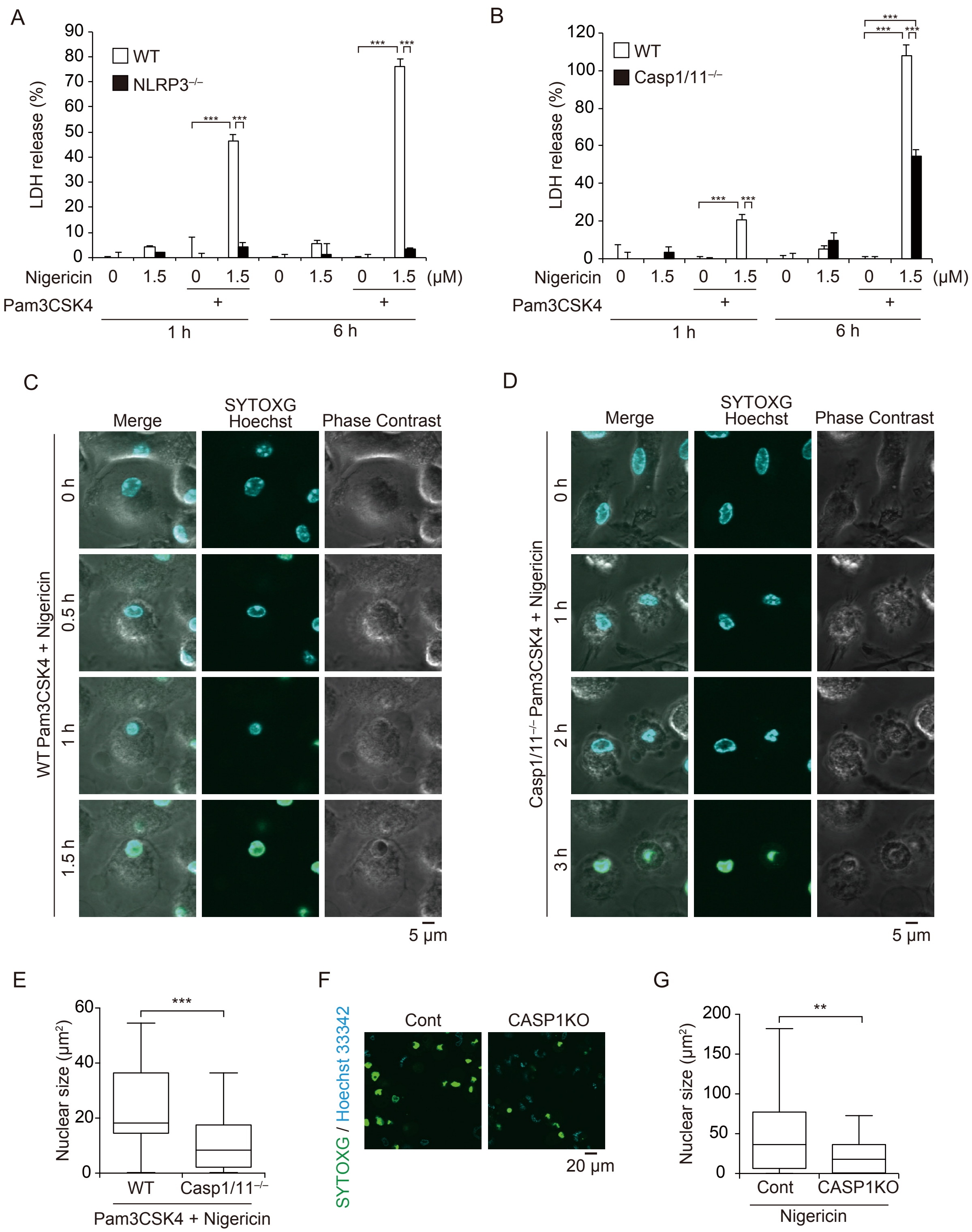

A
LDH release (%)
WT
NLRP3−/−
Nigericin 0 1.5 0 1.5 0 1.5 0 1.5 (μM)
Pam3CSK4 + +
1 h 6 h
B
LDH release (%)
WT
Casp1/11−/−
Nigericin 0 1.5 0 1.5 0 1.5 0 1.5 (μM)
Pam3CSK4 + +
1 h 6 h
C
Merge
SYTOXG Hoechst
Phase Contrast
WT Pam3CSK4 + Nigericin
0 h
0.5 h
1 h
1.5 h
5 μm
D
Merge
SYTOXG Hoechst
Phase Contrast
Casp1/11−/− Pam3CSK4 + Nigericin
0 h
1 h
2 h
3 h
5 μm
E
Nuclear size (μm²)
WT
Casp1/11−/−
Pam3CSK4 + Nigericin
F
SYTOXG / Hoechst 33342
Cont
CASP1KO
20 μm
G
Nuclear size (μm²)
Cont
CASP1KO
Nigericin

Figure S2

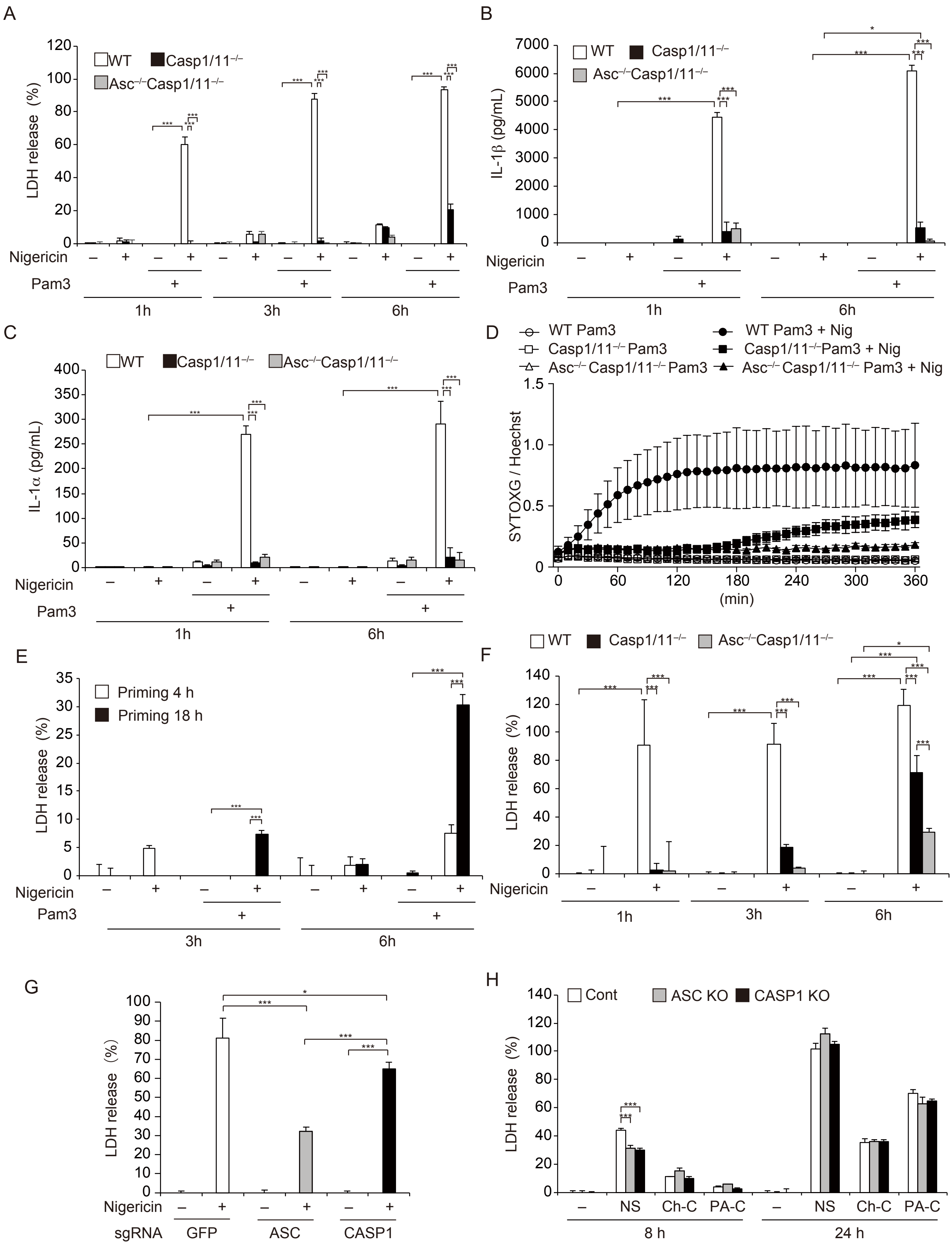
A
LDH release (%)
WT
Casp1/11−/−
Asc−/−Casp1/11−/−
Nigericin
Pam3
1h
3h
6h
B
IL-1β (pg/mL)
WT
Casp1/11−/−
Asc−/−Casp1/11−/−
Nigericin
Pam3
1h
6h
C
IL-1α (pg/mL)
WT
Casp1/11−/−
Asc−/−Casp1/11−/−
Nigericin
Pam3
1h
6h
D
WT Pam3
Casp1/11−/− Pam3
Asc−/− Casp1/11−/− Pam3
WT Pam3 + Nig
Casp1/11−/−Pam3 + Nig
Asc−/− Casp1/11−/− Pam3 + Nig
SYTOXG / Hoechst
(min)
E
LDH release (%)
Priming 4 h
Priming 18 h
Nigericin
Pam3
3h
6h
F
WT
Casp1/11−/−
Asc−/−Casp1/11−/−
LDH release (%)
Nigericin
1h
3h
6h
G
LDH release (%)
Nigericin
sgRNA
GFP
ASC
CASP1
H
Cont
ASC KO
CASP1 KO
LDH release (%)
NS
Ch-C
PA-C
8 h
24 h

Figure S3

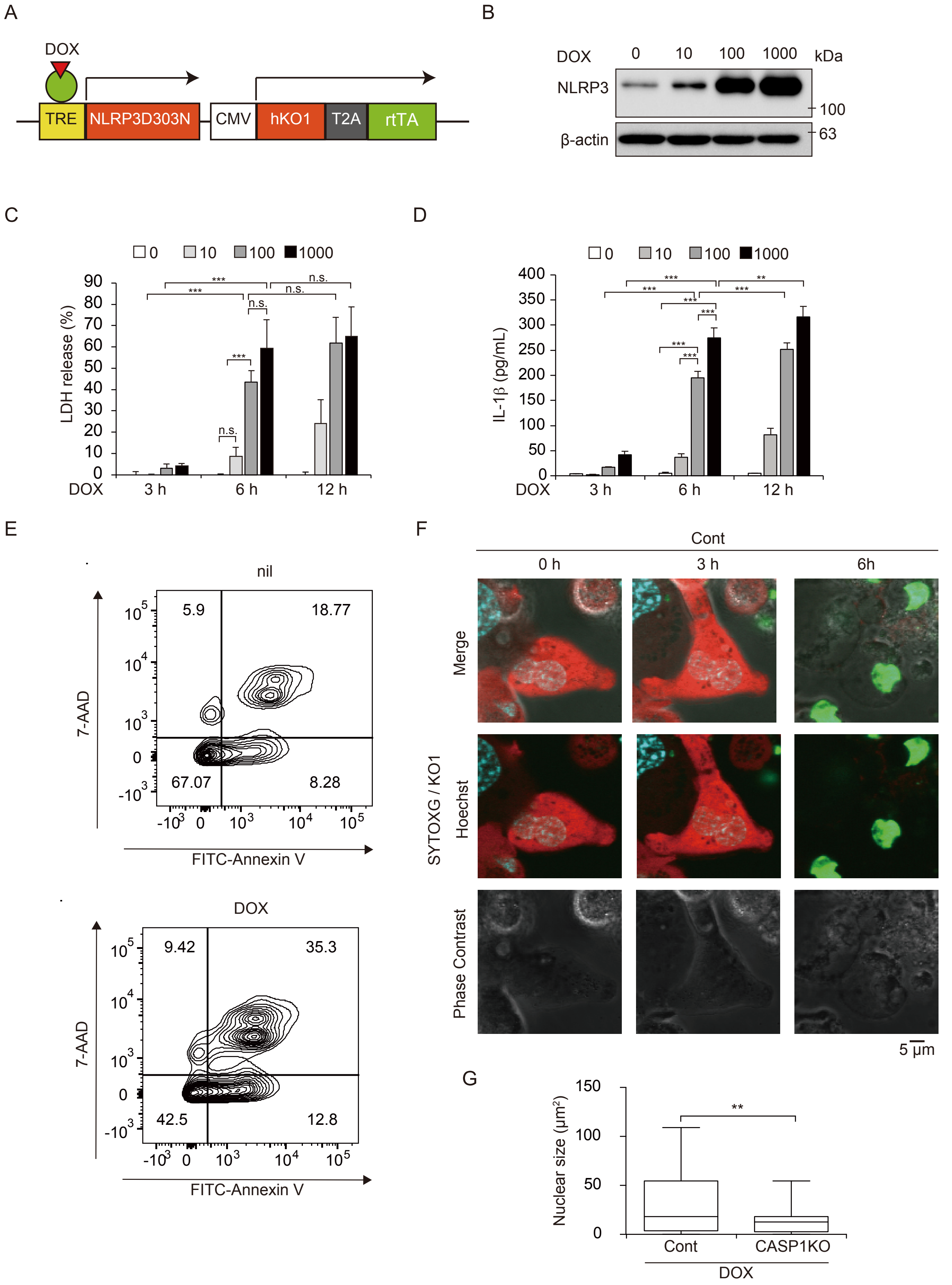

A
DOX
TRE
NLRP3D303N
CMV
hKO1
T2A
rtTA
B
DOX
0
10
100
1000
kDa
NLRP3
100
β-actin
63
C
0
10
100
1000
LDH release (%)
n.s.
***
DOX
3 h
6 h
12 h
D
IL-1β (pg/mL)
**
***
E
nil
7-AAD
FITC-Annexin V
5.9
18.77
67.07
8.28
DOX
9.42
35.3
42.5
12.8
F
Cont
0 h
3 h
6h
Merge
SYTOXG / KO1
Hoechst
Phase Contrast
5 μm
G
Nuclear size (μm²)
Cont
CASP1KO

Figure S4

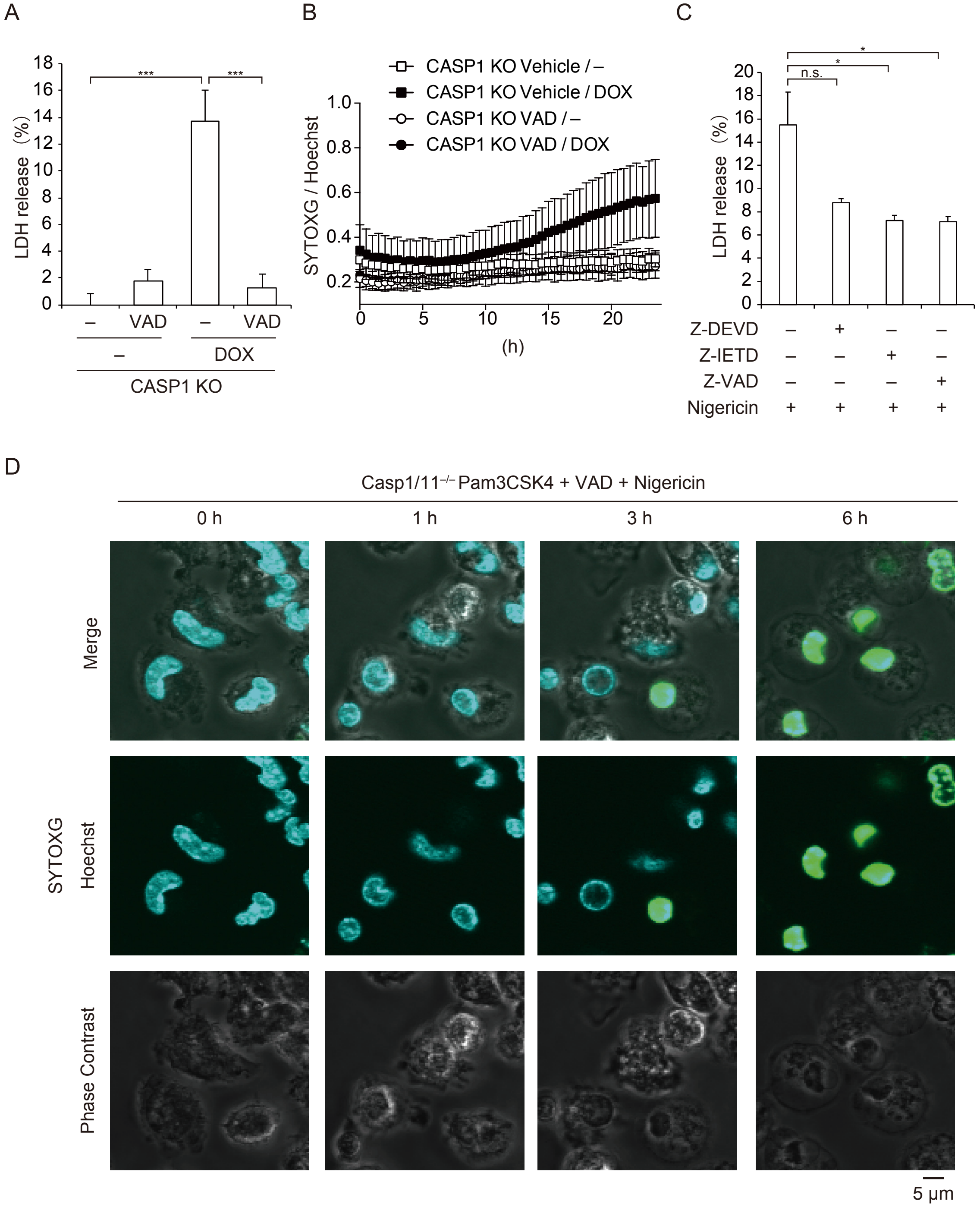
A
18
16
14
12
10
8
6
4
2
0
LDH release (%)
***
***
–
VAD
–
VAD
–
DOX
CASP1 KO
B
CASP1 KO Vehicle / –
CASP1 KO Vehicle / DOX
CASP1 KO VAD / –
CASP1 KO VAD / DOX
SYTOXG / Hoechst
1.0
0.8
0.6
0.4
0.2
0
5
10
15
20
(h)
C
20
18
16
14
12
10
8
6
4
2
0
LDH release (%)
n.s.
*
*
Z-DEVD – + – –
Z-IETD – – + –
Z-VAD – – – +
Nigericin + + + +
D
Casp1/11–/– Pam3CSK4 + VAD + Nigericin
0 h
1 h
3 h
6 h
Merge
SYTOXG
Hoechst
Phase Contrast
5 μm

Figure S5

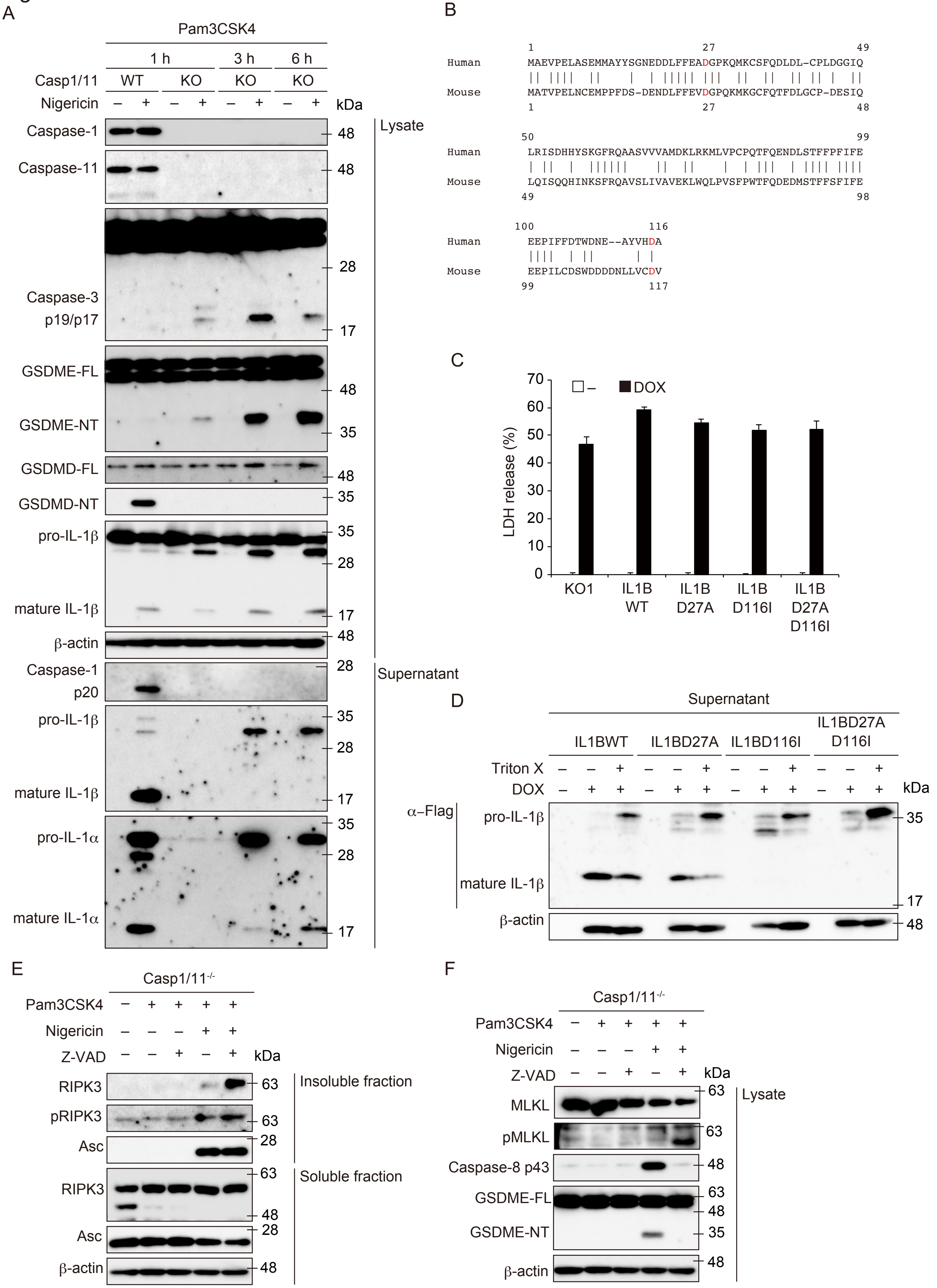
A
Pam3CSK4
1 h
3 h
6 h
Casp1/11
WT
KO
KO
KO
Nigericin
kDa
Lysate
Caspase-1
Caspase-11
Caspase-3
p19/p17
GSDME-FL
GSDME-NT
GSDMD-FL
GSDMD-NT
pro-IL-1β
mature IL-1β
β-actin
Supernatant
Caspase-1
p20
pro-IL-1β
mature IL-1β
pro-IL-1α
mature IL-1α
B
1 27 49
Human MAEVPELASEMMAYYSGNEDDLFFEADGPKQMKCSFQDLDL-CPLDGGIQ
Mouse MATVPELNCEMPPFDS-DENDLFFEVDGPQKMKGCFQTFDLGCP-DESIQ
1 27 48
50 99
Human LRISDHHYSKGFRQAASVVVAMDKLRKMLVPCPQTFQENDLSTFFPFIFE
Mouse LQISQQHINKSFRQAVSLIVAVEKLWQLPVSFPWTFQDEDMSTFFSFIFE
49 98
100 116
Human EEPIFFDTWDNE--AYVHDA
Mouse EEPILCDSWDDDDNLLVCDV
99 117
C
– DOX
LDH release (%)
KO1
IL1B WT
IL1B D27A
IL1B D116I
IL1B D27A D116I
D
Supernatant
IL1BWT
IL1BD27A
IL1BD116I
IL1BD27A D116I
Triton X
DOX
kDa
α–Flag
pro-IL-1β
mature IL-1β
β-actin
E
Casp1/11-/-
Pam3CSK4
Nigericin
Z-VAD
kDa
RIPK3
pRIPK3
Asc
Insoluble fraction
RIPK3
Asc
β-actin
Soluble fraction
F
Casp1/11-/-
Pam3CSK4
Nigericin
Z-VAD
kDa
Lysate
MLKL
pMLKL
Caspase-8 p43
GSDME-FL
GSDME-NT
β-actin

Figure S6

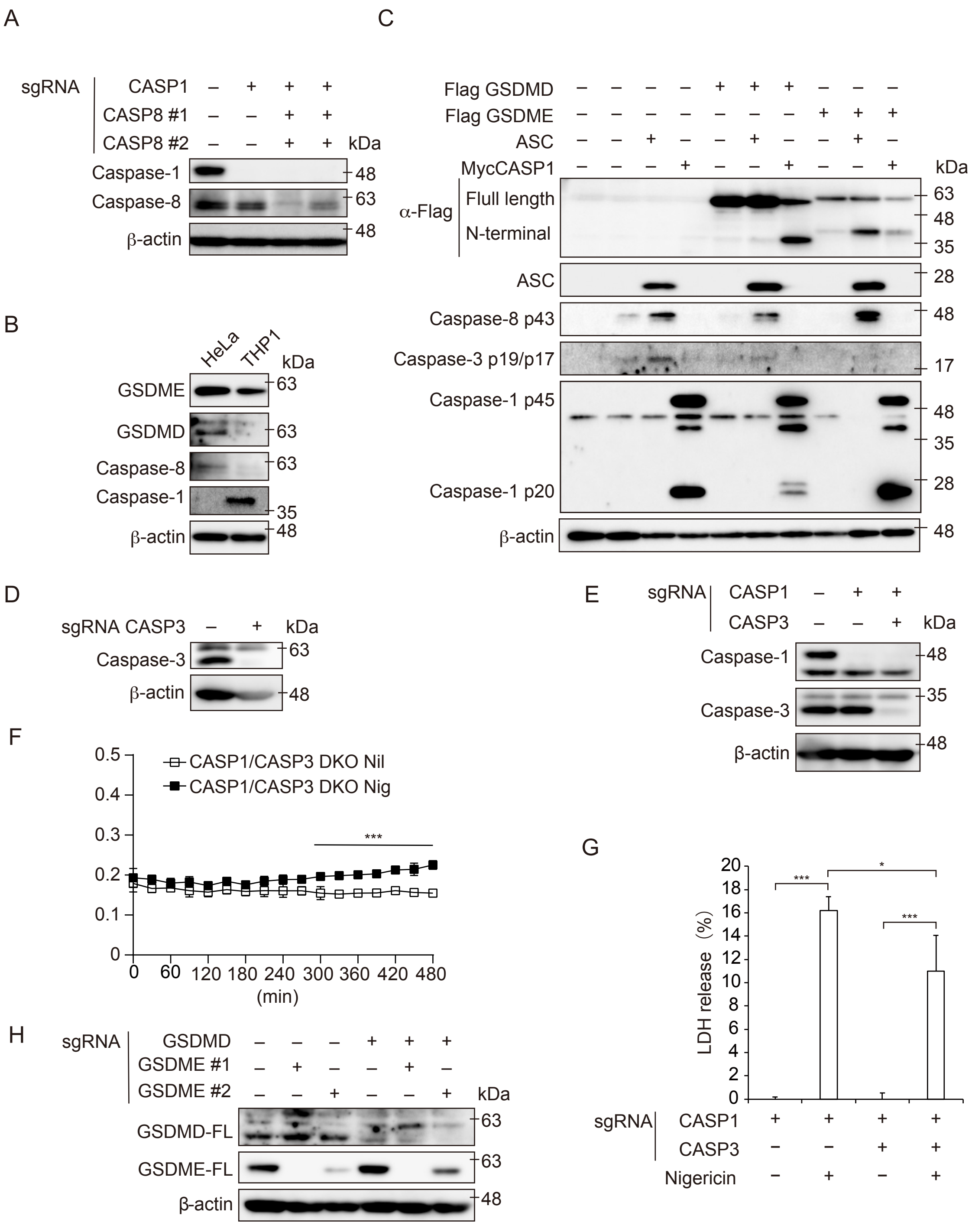
A
sgRNA CASP1 – + + +
CASP8 #1 – – + +
CASP8 #2 – – + + kDa
Caspase-1 48
Caspase-8 63
β-actin 48
B
HeLa THP1 kDa
GSDME 63
GSDMD 63
Caspase-8 63
Caspase-1 35
β-actin 48
C
Flag GSDMD
Flag GSDME
ASC
MycCASP1 kDa
α-Flag Flull length 63 48
N-terminal 35
ASC 28
Caspase-8 p43 48
Caspase-3 p19/p17 17
Caspase-1 p45 48 35
Caspase-1 p20 28
β-actin 48
D
sgRNA CASP3 – + kDa
Caspase-3 63
β-actin 48
E
sgRNA CASP1 – + +
CASP3 – – + kDa
Caspase-1 48
Caspase-3 35
β-actin 48
F
CASP1/CASP3 DKO Nil
CASP1/CASP3 DKO Nig
***
(min)
G
LDH release (%)
*** * ***
sgRNA CASP1 + + + +
CASP3 – – + +
Nigericin – + – +
H
sgRNA GSDMD – – – + + +
GSDME #1 – + – – + –
GSDME #2 – – + – – + kDa
GSDMD-FL 63
GSDME-FL 63
β-actin 48

Figure S7

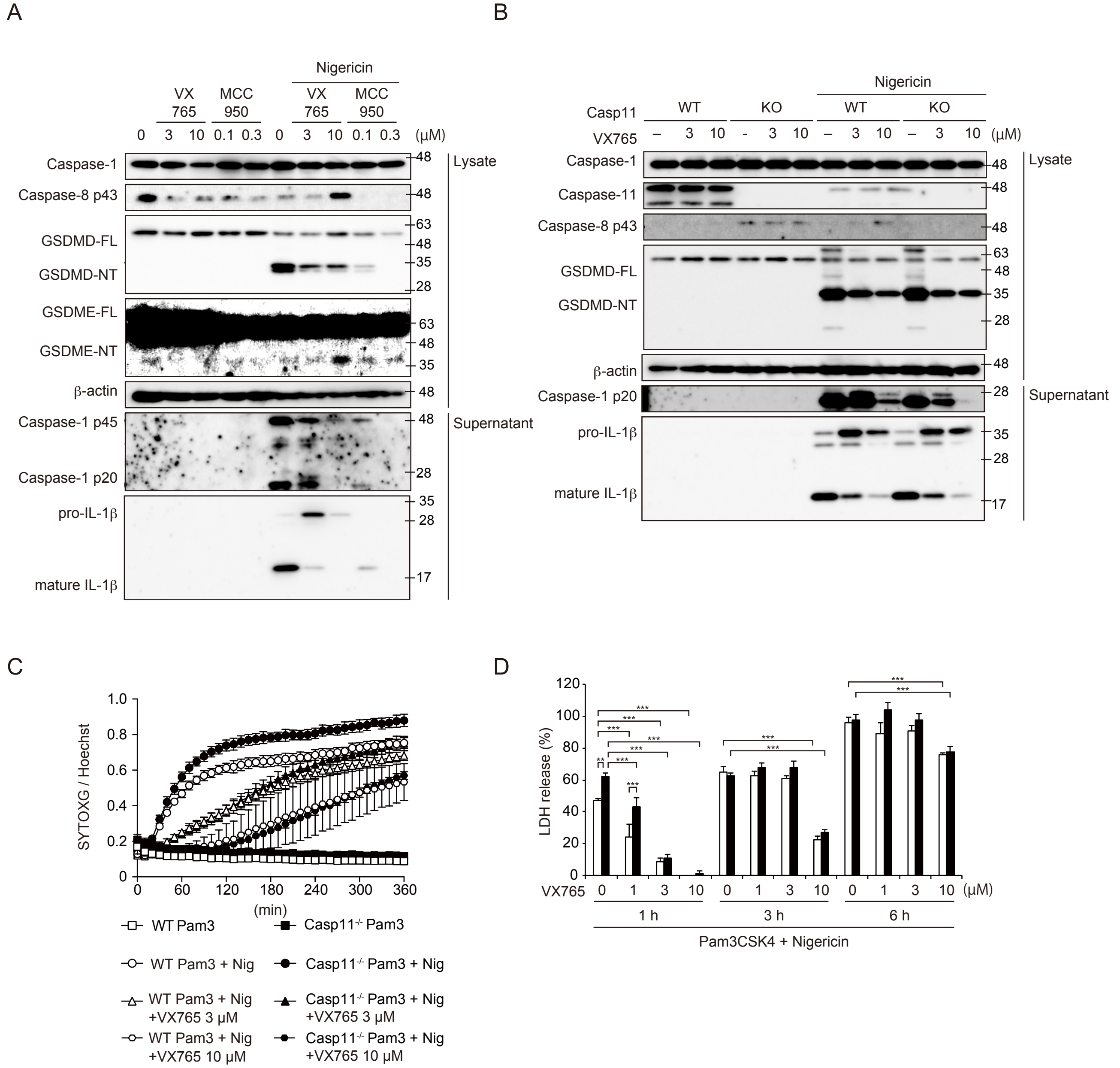
A
B
C
D
Nigericin
VX 765
MCC 950
Caspase-1
Caspase-8 p43
GSDMD-FL
GSDMD-NT
GSDME-FL
GSDME-NT
β-actin
Caspase-1 p45
Caspase-1 p20
pro-IL-1β
mature IL-1β
Lysate
Supernatant
Casp11
WT
KO
VX765
Caspase-11
SYTOXG / Hoechst
(min)
WT Pam3
Casp11-/- Pam3
WT Pam3 + Nig
Casp11-/- Pam3 + Nig
WT Pam3 + Nig +VX765 3 μM
Casp11-/- Pam3 + Nig +VX765 3 μM
WT Pam3 + Nig +VX765 10 μM
Casp11-/- Pam3 + Nig +VX765 10 μM
LDH release (%)
1 h
3 h
6 h
Pam3CSK4 + Nigericin

Figure S8

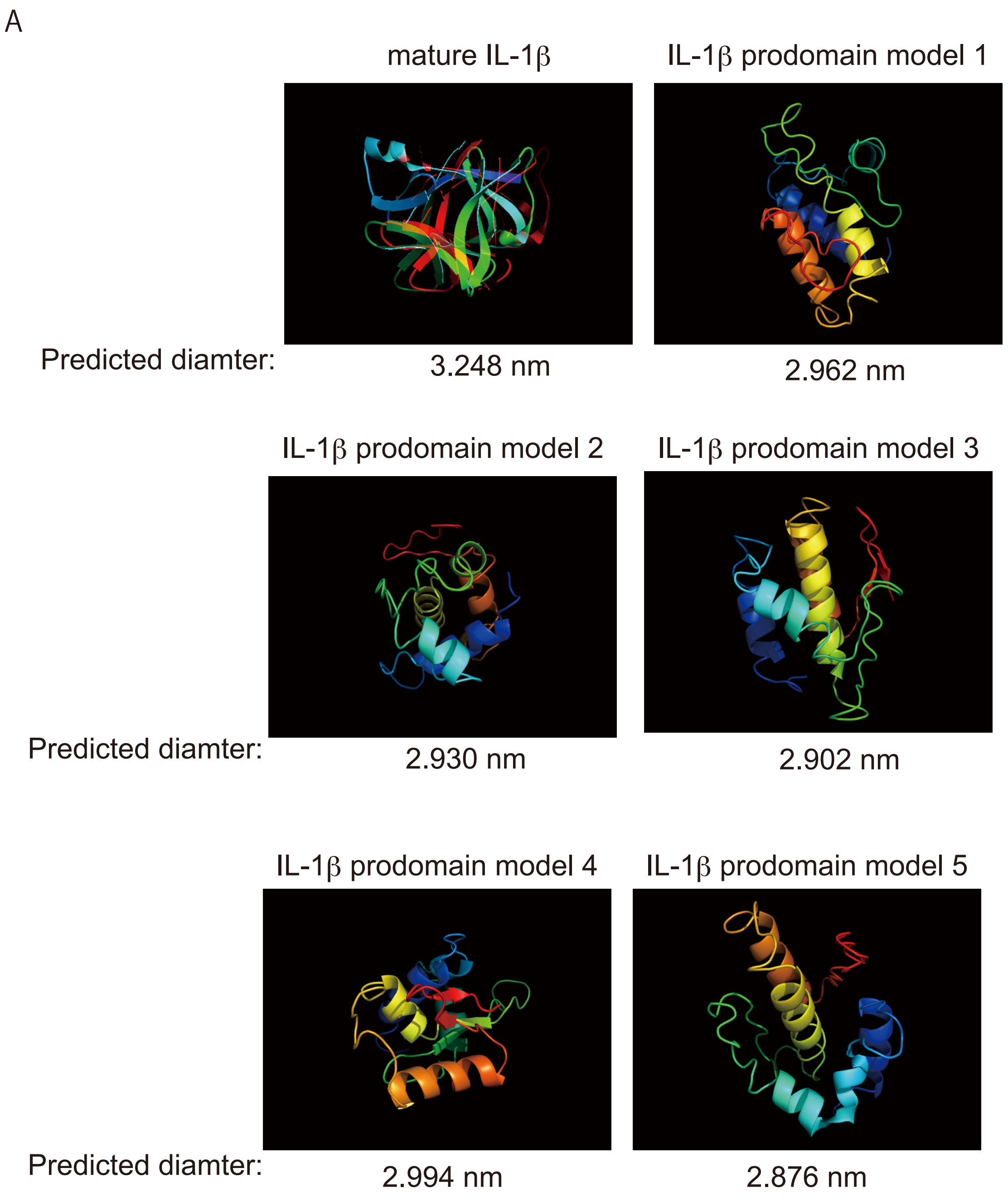
A
mature IL-1β
IL-1β prodomain model 1
Predicted diamter:
3.248 nm
2.962 nm
IL-1β prodomain model 2
IL-1β prodomain model 3
Predicted diamter:
2.930 nm
2.902 nm
IL-1β prodomain model 4
IL-1β prodomain model 5
Predicted diamter:
2.994 nm
2.876 nm
